# Sequences of Two Related Multiple Antibiotic Resistance Virulence Plasmids Sharing a Unique IS*26*-Related Molecular Signature Isolated from Different *Escherichia coli* Pathotypes from Different Hosts

**DOI:** 10.1371/journal.pone.0078862

**Published:** 2013-11-04

**Authors:** Carola Venturini, Karl A. Hassan, Piklu Roy Chowdhury, Ian T. Paulsen, Mark J. Walker, Steven P. Djordjevic

**Affiliations:** 1 School of Chemistry and Molecular Biosciences and Australian Infectious Diseases Research Centre, the University of Queensland, Brisbane, Queensland, Australia; 2 Department of Chemistry and Biomolecular Sciences, Macquarie University, Macquarie Park, New South Wales, Australia; 3 NSW Department of Primary Industries, Camden, New South Wales, Australia; 4 The ithree Institute - Infection. Immunity. Innovation, University of Technology, Sydney, New South Wales, Australia; University of Manchester, United Kingdom

## Abstract

Enterohemorrhagic Escherichia coli (EHEC) and atypical enteropathogenic *E. coli* (aEPEC) are important zoonotic pathogens that increasingly are becoming resistant to multiple antibiotics. Here we describe two plasmids, pO26-CRL_125_ (125 kb) from a human O26:H- EHEC, and pO111-CRL_115_ (115kb) from a bovine O111 aEPEC, that impart resistance to ampicillin, kanamycin, neomycin, streptomycin, sulfathiazole, trimethoprim and tetracycline and both contain atypical class 1 integrons with an identical IS*26*-mediated deletion in their 3´-conserved segment. Complete sequence analysis showed that pO26-CRL_125_ and pO111-CRL_115_ are essentially identical except for a 9.7 kb fragment, present in the backbone of pO26-CRL_125_ but absent in pO111-CRL_115_, and several indels. The 9.7 kb fragment encodes IncI-associated genes involved in plasmid stability during conjugation, a putative transposase gene and three imperfect repeats. Contiguous sequence identical to regions within these pO26-CRL_125_ imperfect repeats was identified in pO111-CRL_115_ precisely where the 9.7 kb fragment is missing, suggesting it may be mobile. Sequences shared between the plasmids include a complete IncZ replicon, a unique toxin/antitoxin system, IncI stability and maintenance genes, a novel putative serine protease autotransporter, and an IncI1 transfer system including a unique shufflon. Both plasmids carry a derivate Tn*21* transposon with an atypical class 1 integron comprising a *dfrA5* gene cassette encoding resistance to trimethoprim, and 24 bp of the 3´-conserved segment followed by Tn*6026*, which encodes resistance to ampicillin, kanymycin, neomycin, streptomycin and sulfathiazole. The Tn*21*-derivative transposon is linked to a truncated Tn*1721*, encoding resistance to tetracycline, via a region containing the IncP-1α *oriV*. Absence of the 5 bp direct repeats flanking Tn*3*-family transposons, indicates that homologous recombination events played a key role in the formation of this complex antibiotic resistance gene locus. Comparative sequence analysis of these closely related plasmids reveals aspects of plasmid evolution in pathogenic *E. coli* from different hosts.

## Introduction

Enterohemorrhagic Escherichia coli (EHEC) and enteropathogenic *E. coli* (EPEC) are important diarrheagenic pathotypes responsible for substantial human morbidity and mortality [1]. Both carry a chromosomally-located island known as the locus of enterocyte effacement (LEE) that produces essential effector molecules required for the formation of characteristic attaching and effacing lesions on gastrointestinal epithelial cells [2]. EHEC are a subset of Shiga toxin-producing *E. coli* (STEC) expressing phage-derived Shiga toxins and accessory virulence factors, including intimin (Eae) and the plasmid-encoded enterohemolysin EhxA, responsible for the development of serious post-infection sequelae, such as haemorrhagic colitis and haemolytic uremic syndrome (HUS) [3]. Although most EHEC infections cause self-limiting bloody diarrhoea, in 5 to 7% of cases patients develop HUS, the leading cause of acute renal failure in children [4].

Ruminants are a key reservoir for both EHEC and atypical EPEC (aEPEC), an emerging cause of diarrhoea in both humans and animals globally [5,6]. More than 400 STEC serotypes have been described many of which are recoverable from faeces [7,8,9]. EHEC serotype O157:H7 is responsible for most cases of HUS particularly in the United States, Japan, Scotland, Canada and England. In the USA, O157:H7 EHEC infection causes approximately 73,000 illnesses resulting in several thousand hospitalizations and over 60 deaths per annum [10]. However, other EHEC serotypes including O26:H11/H- and O111:H8/H2/H- are also responsible for both large and sporadic outbreaks of serious disease worldwide [9,11,12]. EHEC O26:H^-^/H11 is a leading cause of HUS in many European countries [13,14] and has recently been associated with severe paediatric cases [15]. The validity of antibiotic therapy in the treatment of EHEC infection is controversial [16,17] with reports of antibiotics both inducing the SOS response and influencing the stability and subsequent release of Shiga toxin phage [18,19]. Despite these concerns, the German Society for Infection recommended the use of antibiotics for the treatment of patients infected with O104:H4, responsible for the world’s largest HUS outbreak [20].

Multiple antibiotic resistance in EHEC, particularly O157:H7, O26:H-/H11 and O111:H8, is a serious concern [21,22,23]. Genetic elements encoding multiple drug resistance (MDR) are often associated with complex antibiotic resistance gene loci (CRL) comprising mobile genetic elements, often located on transmissible plasmids of the IncI and IncF groups [24,25]. IS*26* in association with Tn*3*-type transposons plays a particularly important role in the evolution of MDR plasmids and chromosomal islands in the *Enterobacteriaceae* [24,26,27,28]. Homologous and site-specific recombination events involving these mobilizable CRL are shaping the rapid evolution of MDR in the gut microflora resulting in the more frequent isolation of complex mosaic plasmid backbones carrying multiple replicons, and antimicrobial drug resistance and virulence genes [24,29].

In a previous study, we isolated multiply resistant EHEC and aEPEC by screening for atypical class 1 integrons where IS*26* abuts a truncated version of the 3´-CS (conserved segment) [30]. Multiply resistant EHEC O26:H- strain O6877, isolated from a patient with bloody diarrhoea, displays resistance to ampicillin (Ap), kanamycin (Km), streptomycin (Sm), sulfathiazole (Su), tetracycline (Tc) and trimethoprim (Tm), is toxigenic for Vero cells, enterohemolytic on washed sheep blood agar and carries Shiga toxin 1 (stx_1_), intimin (*eae*) and enterohemolysin (*ehxA*) genes [26,31]. Virulence genes encoding enterohemolysin (EhxA), a putative adhesin (ToxB), a catalase/peroxidase (KatP), a serine protease (EspP), several proteins involved in biofilm formation (MsbB and ShdA) and a Tn*21* derivative, carrying antibiotic resistance genes encoding resistance to Ap-Km-Sm-Su-Tm, were shown to be located on an 111,481 bp MDR plasmid, pO26-CRL [26]. The Tn*21* derivate transposon houses an atypical integron containing a *dfrA5* cassette, encoding Tm resistance, and a truncated 3´-CS, followed by the complex MDR transposon Tn*6026*, containing *bla*_TEM-1_, *aphA1*, *strAB*, and *sul2*, encoding resistance to Ap, Km/neomycin (Nm), Sm and Su respectively. The modified integron accounts for the antibiotic resistance phenotype of strain O6877 with the notable exception of Tc [26,28]. Strain O6877 was earmarked for molecular characterization as it was representative of the subset of multidrug resistant *E. coli* containing these atypical class 1 integrons [30].

Here, we report the complete sequence of a 125 kb MDR plasmid, identified as pO26-CRL_125_, isolated from human O26:H- strain O6877. Like co-resident plasmid pO26-CRL (renamed here as pO26-CRL_111_), pO26-CRL_125_ confers resistance to Ap, Km, Sm, Su and Tm but it also encodes resistance to Tc. We also fully sequenced a 115 kb plasmid (pO111-CRL_115_) from O111 aEPEC strain D275, isolated from a bovine with gastrointestinal disease. pO111-CRL_115_ also shares the unique molecular signature created by IS*26*-mediated deletion affecting the structural integrity of the 3ʹ-CS. Our analyses show that plasmids pO26-CRL_125_ and pO111-CRL_115_ are essentially identical. These two plasmids were recovered from serologically different *E. coli*, belonging to different pathotypes, isolated from different animal hosts, yet remarkably share identical genetic architecture with substantial sequence similarity. Analyses of their minor sequence differences revealed several important aspects of *in vivo* plasmid evolution in pathogenic *E. coli* from different hosts.

## Materials and Methods

### Bacterial strains and plasmids

EHEC O26:H- strain O6877 was originally isolated in 1998 [31] and carries two MDR plasmids, pO26-CRL_111_ described earlier [26] and pO26-CRL_125_ (described here). Strain O6877 and O111 aEPEC strain D275 (isolated between 1999 and 2002) were part of a larger collection of 512 serologically diverse MDR *E. coli* including aEPEC, STEC and EHEC of human and bovine origin that were screened for the presence of class 1 integrons [30]. Plasmids pO26-CRL_125_ and pO111-CRL_115_ were isolated from O6877 and D275 respectively and sequenced.

### Plasmid isolation

Plasmids from *E. coli* strains O6877 and D275 were conjugated with *E. coli* DH5α as previously described [32]. Gel electrophoresis of plasmid preparations showed that the wildtype strains carried several plasmids of different molecular size. As this potentially complicates sequencing studies, purified plasmid preparations from each strain were used in transformation using *E. coli* TOP10 as recipient following standard protocols (Invitrogen, Mulgrave, Vic, Australia). Transconjugants and transformants were tested for resistance to appropriate panels of antibiotics as described previously [26] and examined by gel electrophoresis to ensure each carried a single plasmid species.

### Sequencing

Plasmids were isolated from *E. coli* DH5α or TOP10 hosts using the plasmid Midi Prep extraction kit (Qiagen, Doncaster, Vic, Australia). Plasmid sequencing was performed using Roche 454 GS FLX technology at the Ramaciotti Center for Gene Function Analysis. MDR plasmids were multiplexed with equal total DNA concentrations providing approximately 25 to 700x sequence coverage. Plasmids pO26-CRL_125_ and pO111-CRL_115_ were sequenced to 49 and 25x coverage, respectively. Plasmid sequences were assembled *de novo* using the Newbler v2.3 software package (454 Life Sciences, a Roche company, Branford, CT, USA). Contigs were broken by long repeat sequences, typically IS*26* and were assembled into a single sequence by PCR between adjacent contigs. Contaminant chromosomal sequences were present at significantly reduced coverage, typically less than 3x. Plasmid contigs were initially annotated automatically using the RAST server [33]. Subsequently, the annotation was manually curated.

### Sequence analysis

For sequence analysis and manual annotation, the BLAST algorithm [34] (www.ncbi.nlm.nih.gov/BLAST), insertion sequence (IS) finder [35] (www-is.biotoul.fr), open reading frame (ORF) finder (www.ncbi.nlm.nih.gov/projects/gorf), and the VectorNti software program (Invitrogen, Mulgrave, Vic, Australia) were utilized. EMBOSS Needle alignment tool (http://www.ebi.ac.uk/Tools/psa/emboss_needle/) and ClustalO [36] (http://www.ebi.ac.uk/Tools/msa/clustalo) were used for nucleotide and protein sequence comparison. Protein sequences were characterized using the Pfam database (http://pfam.sanger.ac.uk/) for annotation of protein function. Circular representations of the plasmids, including comparisons to related plasmids were made using CGview [37].

### Stability assays

Stability experiments were performed essentially as previously described [38]. Briefly, overnight cultures in LB Tc (20 μg/ml) of transformed *E. coli* TOP10 strains harboring pO26-CRL_125_ or pO111-CRL_115_ (37 °C with shaking) were washed to remove the antibiotics and resuspended in saline. Each day 4.88 μl from fresh cultures were transferred to 5 ml LB in order to obtain about 10 generations per 24 h growth cycle (37 °C with shaking at constant speed). At selected time points, the growing cultures were plated both onto LB agar and LB agar supplemented with Tc (20 μg/ml) and incubated overnight at 37 °C. Determining the fraction of plasmid free cells in the population was done by calculating the ratio between the total number of cells (LB only) and the number of plasmid-containing cells (LB Tc). For each strain, stability experiments were performed in triplicate over 80 generations. The data were plotted using the Graph Pad Prism software (Graph Pad Software, Inc., San Diego, CA, US).

### Conjugal transfer of pO111-CRL_115_ and pO26-CRL_125_ into JM109 Rif^r^Nal^r^ *E. coli*

Conjugal capacity of pO26-CRL_125_ and pO111-CRL_115_ was tested by mating the *E. coli* TOP10 transformants with JM109 Rif^r^Nal^r^, a recipient rifampicin resistant *E. coli* JM109 made resistant to nalidixic acid (Nal) by multiple subculture on selective LB agar with increasing Nal concentrations (2 to 30 μg/mL). To confirm conjugation frequencies, a second assay was carried out following the same protocol with the inclusion in the mating mixture of *E. coli* HB101 containing the chloramphenicol resistant conjugative helper plasmid pRK600 [39] (not shown). Transconjugants were plated onto LB agar co-selecting for both the MDR plasmid (Ap, 100 μg/ml) and the recipient JM109 Rif^r^Nal^r^ (Nal 30 μg/ml). Each mating was also plated onto LB agar selecting for the recipient only (Nal 30 μg/ml). Donor only and recipient only negative controls were also plated onto the same media. The conjugal transfer frequency was calculated as the number of transconjugants per number of recipient cells. Transconjugants were tested for resistance on media supplemented with the following antibiotics: Rif (100 μg/ml), Ap (32 μg/ml), Km (10 μg/ml), Tc (20 μg/ml), and Su (550 μg/ml).

### Nucleotide sequences accession number

The complete nucleotide sequences for pO26-CRL_125_ and pO111-CRL_115_ have been submitted to the GenBank database under accession numbers KC340959 for pO111-CRL_115_ and KC340960 for pO26-CRL_125_.

## Results

### Sequence analysis of pO26-CRL_125_ and pO111-CRL_115_

**Figure 1:**
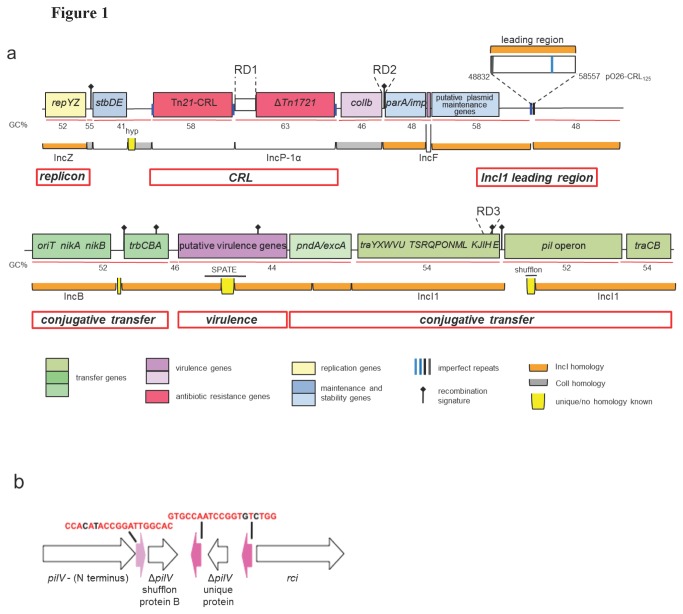
Main distinguishing features of plasmids pO26-CRL_125_ and pO111-CRL_115_. **a**. Schematic visualization of gene arrangement in pO26-CRL_125_ and pO111-CRL_115_. Genes with known function and competency modules are indicated by name. Overall mean G + C content is indicated in percentages (GC%). Highest sequence homology to specific Inc groups is indicated by labeled horizontal brackets. RD1, RD2 and RD3 indicate the position of the regions of sequence where pO26-CRL_125_ and pO111-CRL_115_ differ substantially. Potential recombination signatures were identified by BLAST analysis. Regions of interest in plasmid architecture are labeled with red boxes (bold italics). b Depiction of the unique shufflon found in pO111-CRL_115_ and pO26-CRL_125_. This module presents a single invertible fragment selecting for two possible PilV C-termini. The sequence of the characteristic 19 bp repeats, thought to be involved in the site-specific recombination events leading to *pilV* inversions, is shown. Drawings not to scale.

**Figure 2:**
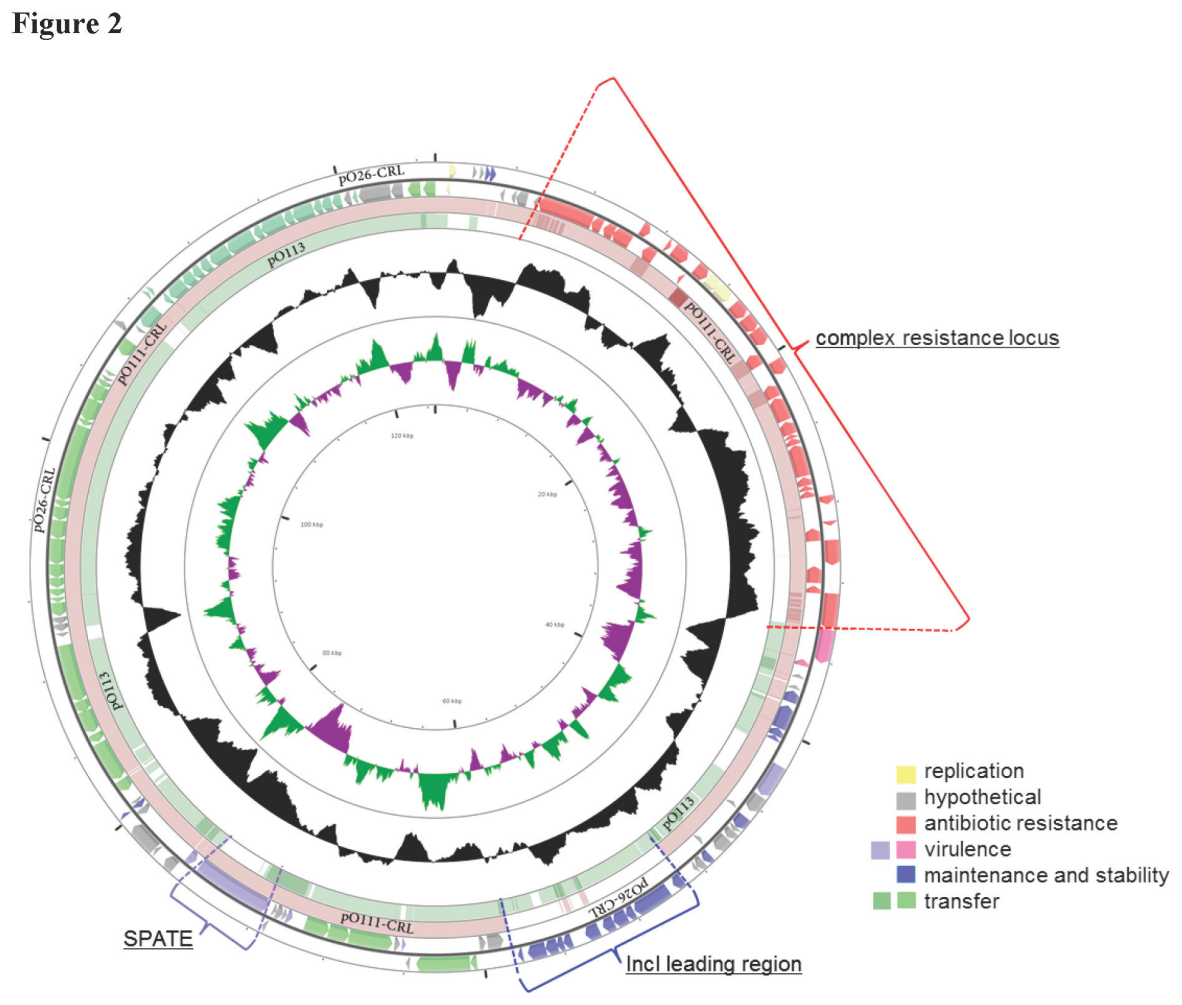
Circular representation of plasmid pO26-CRL_125_. The outer two circles (+ and − strands) show the coding sequences of plasmid pO26-CRL_125_, colored according to broad function. The two inner circles represent BLAST comparisons to pO111-CRL_115_ (pale maroon circle) and EHEC plasmid pO113 (GenBank AY258503) (pale green innermost circle), where the color shading is reflective of the similarity in nucleotide sequence. pO111-CRL_115_ contains the same complex multidrug resistance locus encoding resistance to seven antibiotics and comprising both a Tn*21* derivative transposon and a truncated Tn*1721* (red block arrows). Genes associated with virulence (purple/pink arrows) are almost all clustered in one position. Operons involved in conjugal transfer mechanisms (green arrows) show high homology to the same modules in pO113. The main regions of interest in the architecture of these plasmids (multi-resistance region, leading region and SPATE) are labeled with brackets. Figure prepared using CGView [37].

pO26-CRL_125_ and pO111-CRL_115_ comprise 124,908 bp and 115,452 bp respectively. Both display an average G + C content of about 53%, around 2.5-3% higher than the G + C content of sequenced *E. coli* O26 and O111 chromosomes [40]. The general structure of pO26-CRL_125_ and pO111-CRL_115_ is shown in Figure 1. The two plasmids are almost identical except for: i) a 9,726 bp fragment found only in pO26-CRL_125_ (leading region), ii) differences in the number of repeats in two unrelated repeat regions (RD1 and RD2), iii) a 135 bp deletion in the *traH* open reading frame (orf) in pO26-CRL_125_ (RD3), iv) one small indel and two point mutations (Figure 1; Table 1). pO26-CRL_125_ and pO111-CRL_115_ contain 147 and 136 predicted orfs respectively (Table S1). The plasmid backbones encode genes for replication, stability and maintenance, and conjugal transfer (Figure 2; Table S1), and display a mosaic structure where modules characteristic of plasmids belonging to different incompatibility types are assembled in a novel arrangement. Large portions of the backbones share high sequence identity (>95%) with IncI EHEC plasmids, such as IncI1 pO113 (GenBank AY258503) (Figure 2), with some modules showing homology to specific elements of plasmids belonging to incompatibility groups IncZ (pIE545; GenBank M93064.1), IncB (p3521; GenBank GU256641) and IncP-1α (pBS228; GenBank NC_008357) (Figure 1). In this mosaic backbone structure, divergent G + C content for separate regions suggests assembly by multiple horizontal gene transfer events (Figure 1). The accessory gene load in both plasmids consists of a CRL containing derivate Tn*21* and Tn*1721* transposons, and a virulence module, encoding putative virulence factors, including a novel serine protease autotransporter of *Enterobacteriaceae* (SPATE) (Figure 2).

**Table 1:** Sequence differences between *E. coli* plasmids pO26-CRL_125_ and pO111-CRL_115_.

Label*	**Gene/Feature**	**pO111-CRL_115_ nt^+^ position**	**pO26-CRL_125_ nt^+^ position**	**Modification pO111-CRL_115_/pO26-CRL_125_**
RD1	*oriV*-IncP repeat region	28488 - 28648	28488	+ / - 160 bp
RD2	8 bp tandem repeats	37664	37504 - 37520	- / + 16 bp
mut 1	non coding	40761	40617	C / T
mut 2	*hap* (silent)	45075	44931	T / C
LR	IncI1 leading region	48975	48832 - 58557	- / + 9,726 bp
RD3	*traH* (in frame deletion)	94053 - 94188	103633	+ / - 135 bp
indel	non coding	97000	106446 - 106456	- / + 10 bp

* RD, region of difference; LR, leading region; mut, point mutation; indel, insertion-deletion. ^+^nt, nucleotide.

### IncZ and IncQ replicons

**Figure 3:**
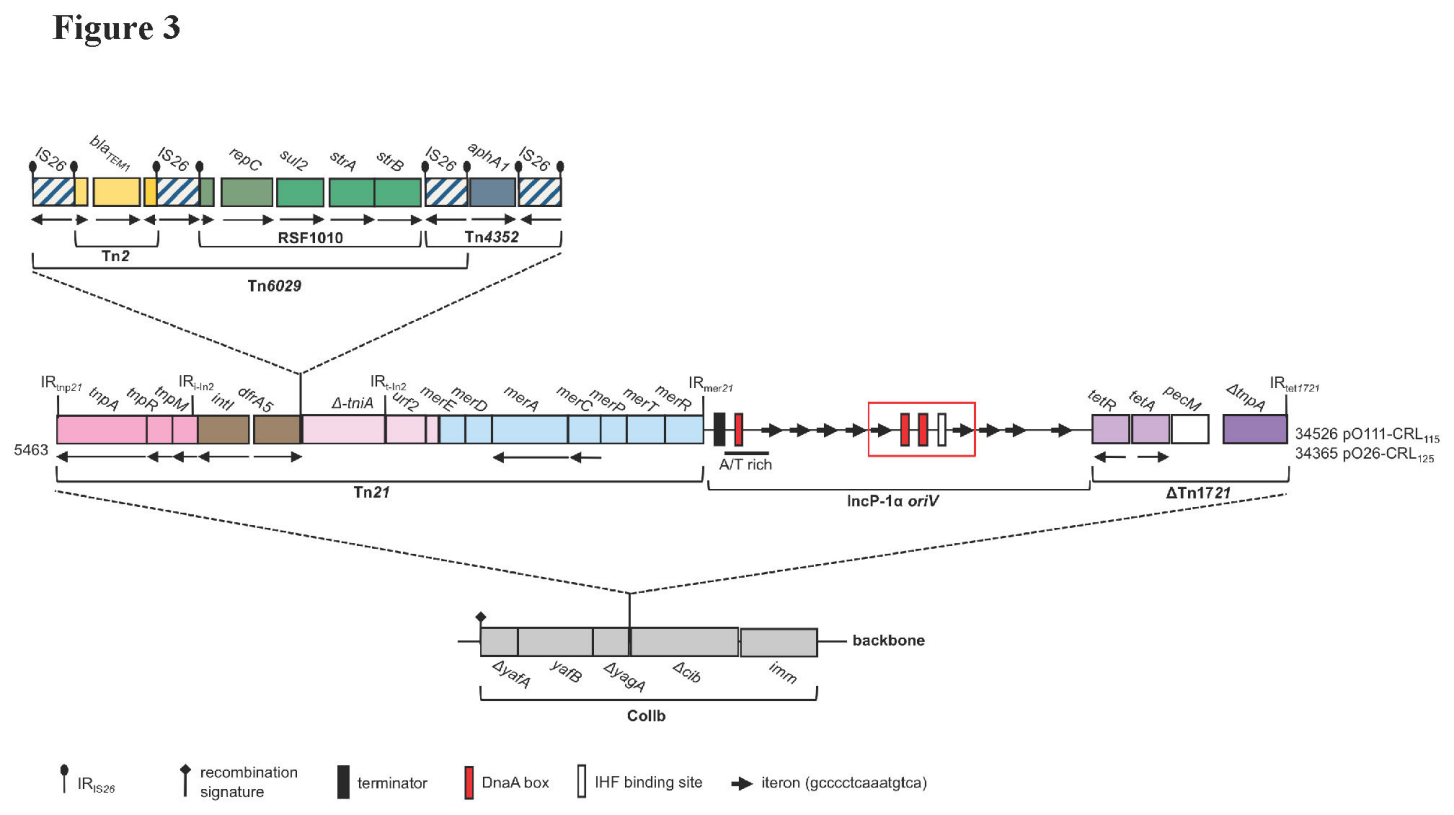
Map of the complex multiple resistance locus (CRL) of pO26-CRL_125_ and pO111-CRL_115_ and schematic representation of its insertion site within the *col* operon. The CRL comprises a Tn*21* derivate, encoding resistance to six antibiotics - *aphA1* (kanamycin and neomycin), *strA-strB* (streptomycin), *sul2* (sulfathiazole), *bla*_TEM-1_ (ampicillin), and *dfrA5* (trimethoprim) - and to mercuric chloride (*mer* operon), and a truncated Tn*1721* (ΔTn*1721*) transposon encoding *tetA*(A) (resistance to tetracycline). The Tn*21* derivative contains a modified class 1 integron, carrying the *dfrA5* cassette, but missing the 3´-CS due to IS*26*-mediated insertion of transposon Tn*6026*. Tn*6026* harbors a copy of Tn*6029*, comprising a truncated Tn*2* with *bla*_TEM-1_ and an RSF1010 segment with *strA-strB* and *sul2*, and a copy of Tn*4352* carrying *aphA1*. In pO111-CRL_115_, Tn*21* and ΔTn*1721* are separated by a region identical to the *oriV* of the IncP-1α pBS228 (GenBank NC_008357). IncP plasmids have a very specific and well characterized *oriV*, comprising a G/C rich region, an A/T rich region, several tandem direct repeats (iterons) and host factors (DnaA, IHF) binding motifs . In pO111-CRL_115_, as in pBS228, one IHF and three DnaA (DnaA boxes) binding sites plus nine iterons are present. In pO26-CRL_125_, part of this *oriV* sequence, with iterons 5 and 4, two DnaA boxes and the IHF binding motif, is deleted (red frame). The boundaries of the CRL components are labeled with brackets. The insertion point of the multiple resistance locus within the *colIb* operon is shown in the figure. Transcriptional direction is indicated by arrows. Δ denotes a partially deleted or truncated gene. IR denotes inverted repeats: IR_IS26_, inverted repeats of IS*26*; IR_t-In2_ and IR_i-In2_ inverted repeats flanking the class 1 integron found within transposon Tn*21*; IR_*mer21*_, 38 bp inverted repeat of transposon Tn*21* adjacent to the *merR* gene; IR_*tnp21*_, 38 bp inverted repeat of transposon Tn*21* adjacent to the *tnpA* gene; IR_*tet1721*_, 38 bp inverted repeat of transposon Tn*21* adjacent to the *ΔtnpA* gene. Drawing not to scale.

pO26-CRL_125_ and pO111-CRL_115_ contain two separate replication regions, a complete IncZ replicon identical to that of pIE545 (GenBank M93064.1) from *Klebsiella pneumoniae* and a partial IncQ replicon within the Tn*21* derivative transposon in the antibiotic resistance module (Figure 1; Figure 3). The IncZ replicon contains genes coding for RepZ and RepY proteins and an RNAI encoding sequence with homology to that of other plasmids of the IncI complex (Table S1; Figure S1a). Inc RNAI encodes antisense RNA for *repYZ* mRNA and is one of the elements responsible for plasmid incompatibility [41,42]. IncZ plasmids are compatible with IncI1 and IncK plasmids but incompatible with IncB plasmids [41]. Consistent with these reports, IncZ plasmid pO26-CRL_125_ (this study) and IncI1 plasmid pO26-CRL_111_ [26] are co-resident in O26:H- EHEC strain O6877 and appear to be stably maintained. In both pO26-CRL_125_ and pO111-CRL_115_, the defective IncQ replicon, comprising the *repC* gene and a partial *repA* sequence, is identical to the IncQ replicon in the Tn*21* derivative transposon of plasmid pO26-CRL_111_ (Figure 3).

The sequence separating the IncZ *oriV* from the downstream CRL shows homology to ColIb plasmids sequence (99%), except for a 3 kb fragment with low G + C content (41%) containing five orfs with little or no nucleotide homology to known sequences (Figure 1; Table S1). A BLASTn search against sequences deposited in the NCBI database revealed two gaps in alignment bordering the 5´ and 3´ flanks of this intervening sequence likely representing DNA recombination signatures. Two of these orfs encode a putative novel StbD/E type II antitoxin/toxin system with homology to the RelB/E toxin-antitoxin stability systems of *E. coli* and other Gram-negative species [43,44]. The StbD antitoxin partner belongs to the PHD antitoxin family but showed no specific amino acid homology to the RelB component, while the predicted StbE protein showed 80 to 95% identity with representatives of the characterized cytotoxic translational repressor RelE of *E. coli* [43].

### IncI modules form the majority of the plasmid backbone

The backbone of pO111-CRL_115_ and pO26-CRL_125_ contains plasmid maintenance, stability and transfer modules characteristic of IncI plasmids. The maintenance and stability modules of the two plasmids are identical (Figure 2) except for a difference (RD2) in the number of contiguous repeats (8 bp; *aacaagat*) found in a set between the *col* operon and the *parA* gene (Figure 1; Table 1). Sequence comparison with other IncI plasmids in the NCBI database identified alignment gaps in the RD2 region, indicating a potential recombination hotspot. The transfer modules in pO26-CRL_125_ and pO111-CRL_115_ are also virtually identical and contain type IV conjugative transfer operons sharing extensive nucleotide sequence identity (95-100%) with the transfer regions of pO113 (GenBank AY258503) (Figure 2). These modules comprise the *traABC* regulatory genes (~4 kb), the *pil* operon for thin pilus biogenesis (*pilI* to *pilV*; ~13 kb), and *trb*/*tra* genes for conjugal transfer (~29 kb). The *oriT* region, including the *nikA* and *nikB* genes and the 85 bp *oriT* sequence identical to the *oriT* of IncB plasmid p3521 (GenBank GU256641; Figure 1), was located in close proximity to the *trb* operon as in other IncI plasmids (R64 GenBank AP005147; pO113 GenBank AY258503; ColIb-P9 GenBank NC_002122.1). The *oriT* specific sequence with its two sets of imperfect repeats was identified by comparison with the well characterized *oriT* of IncI1 plasmid R64 [45] (Figure S1b). The *tra* operon presents the same gene arrangement observed in pO113 except for 1 kb of sequence between *traU* and *traT* displaying no nucleotide identity with other IncI plasmids. The *traH* sequence of pO111-CRL_115_ is identical to that in pO113 and unique to these plasmids. In pO26-CRL_125_, a 135 bp deletion (45 amino acids) was identified in this orf (RD3; Table 1). This deletion does not disrupt the reading frame in the putative *traH* gene, therefore a functional conserved lipoprotein product can still be expressed in both plasmids.

The nucleotide sequence of the shufflon recombinase that follows the *tra* operon presents no homology to pO113 but is almost identical (92%) to a gene found in a phage sequence from *Salmonella enterica* serovar Hadar ICESe4 (GenBank FR686852). The unique shufflon of pO26-CRL_125_ and pO111-CRL_115_ consists of a single invertible segment (Figure 1b) that represents the 3´ variable portion of the PilV adhesin. Shufflons previously described in IncI plasmids contain four (A, B, C, D) or three 3´-terminal segments (A, B, and C or D) of the *pilV* orf [42,46], while the pO111-CRL_115_ and pO26-CRL_125_ shufflon contains only a B homologous segment and a second region with unique nucleotide sequence encoding an homolog of shufflon protein C (Figure 1b; Table S1). The only other shufflons presenting a single invertible portion have been described in *Salmonella enterica* serovar Typhi [46] and in *S. enterica* serovar Hadar ICESe4 (GenBank FR686852).

### Structure of the complex antibiotic resistance module

Plasmids pO26-CRL_125_ and pO111-CRL_115_ were isolated from pathogenic *E. coli* of human and bovine origin by PCR amplifying the 3´-CS boundary of atypical class 1 integrons. This PCR, which has one primer in *intI1* and another in IS*26*, produced identical 848 bp amplicons indicating that both these plasmids carried a similar derivative Tn*21* transposon to that previously found in O26:H- EHEC strain O6877 [26,30]. pO26-CRL_125_ and pO111-CRL_115_ harbour identical CRL comprising a derivate Tn*21* transposon and a truncated version of Tn*1721* (ΔTn*1721*) separated by a IncP-1α *oriV* sequence (Figure 3). The Tn*21* derivative transposon in these two plasmids shares 100% sequence identity with the Tn*21* derivate in pO26-CRL_111_ (GenBank GQ259888). The mercury resistance module (*merRTPCAD*) and the complete Tn*21* transposition module (*tnpA*, *tnpR*, and *tnpM*) frame a central antibiotic resistance gene cluster comprising a modified class 1 integron. The integron carries a *dfrA5* resistance gene cassette, encoding Tm resistance, and Tn*6026*. Tn*6026* contains *bla*_TEM-1_, *strAB*, *sul2* and *aphA1*, encoding resistance to Ap, Sm, Su, Km/Nm respectively (Figure 3) [26,28]. Tn*6026* lies precisely 24 bp downstream of the beginning of the 3´-CS in both pO26-CRL_125_ and pO111-CRL_115_ as it does in pO26-CRL_111_ [26].

In pO111-CRL_115_, a region encoding an A/T rich segment and nine tandem repeats, identical to the vegetative origin of replication (*oriV*) of the IncP-1α plasmid pBS228 of *Pseudomonas aeruginosa* (GenBank NC_008357) [47], separates the *mer* module of the Tn*21* derivative transposon from ΔTn*1721* (Figure 3). The *oriV*-∆Tn*1721* sequence displays a G + C content of approximately 63% suggesting it was acquired by lateral transfer. IncP plasmids possess a very specific and well characterized *oriV* (Figure 3) [48]. A functional IncP replicon depends on the interaction between the *oriV* locus and the *trfA* gene products [48], but *trfA* was not present in either pO111-CRL_115_ or pO26-CRL_125_. In pO26-CRL_125_, the IncP-*oriV* sequence is identical to that of pO111-CRL_115_ except for 160 bp of missing sequence, containing two DnaA binding sites, one IHF binding moiety and iterons five and four (RD1) (Figure 3). In both pO26-CRL_125_ and pO111-CRL_115_, the junction between *oriV* and ΔTn*1721* is identical to that found in pBS228 (GenBank NC_008357; Figure 3). Also, the *tetR* and *tetA*(A) genes for tetracycline resistance and the *pecM* orf within ΔTn*1721* are identical to both the Tn*1721* prototype sequence (GenBank X61367) [49] and the same genes found in pBS228. Unlike pBS228, however, in both plasmids a truncated version of the Tn*1721 tnpA* gene (∆*tnpA*) is present. Database searches indicated that sequence identical to the entire ΔTn*1721* module is specifically found in IncP-1β plasmid pB10 (GenBank NC_004840) and in IncN plasmid pRSB201 (GenBank JN102341.1), both recovered from wastewater [50,51].

In pO26-CRL_125_ and pO111-CRL_115_, the 5´ and 3´ sequences flanking the CRL were virtually identical to orfs associated with the colicin operon in IncI plasmids (98 to 100% nucleotide identity to ColIb-P9 and pO113) (Figure 1; Figure 3). Comparative analysis with the homologous region of ColIb plasmids showed deletion of the 3´ end of the conserved *yagA* orf of unknown function, and of the 5´ end of the adjacent *colIb* gene, responsible for the production of colicin Ib (Figure 3). Insertion of Tn*21*-RD1-∆Tn*1721* in the *col* operon is unique to pO26-CRL_125_ and pO111-CRL_115_. However, in other IncI plasmids, IS and other mobile genetic elements are known to insert in this same location [42,52]. No identical matches to either the CRL in pO111-CRL_115_ or in pO26-CRL_125_, with the iteron deletions, were found in public databases. However, the arrangement of these CRL is consistent with observations that the *oriV*-*trfA* junction acts as a hot spot for the insertion of antibiotic resistance transposons and IS in the IncP backbone [53,54]. In pO26-CRL_125_ and pO111-CRL_115,_ target repeats characteristic of Tn*21* or Tn*1721* transposition are missing at the CRL insertion site. This indicates that homologous recombination events may have played here a role in the formation of the Tn*21*-RD1-∆Tn*1721* CRL.

### Virulence module: novel serine protease autotransporter sequence

pO26-CRL_125_ and pO111-CRL_115_ contain several genes encoding both putative and established *E. coli* virulence factors (Figure 2; Table S1). An almost complete *col* operon, encoding bacteriocidal properties as well as host-specific colicin immunity [55], is found immediately adjacent to ∆Tn*1721* (Figure 1; Figure 3). In both plasmids, five genes, with putative roles in virulence, were identified clustered together in the region between the *trb* and *tra* operons as in EHEC plasmid pO113 [56] (Figure 1; Figure 2). Among these is a 4,089 bp orf that encodes a novel SPATE. Sequence analysis shows that the putative protein product displays all the features characteristic of SPATEs [57], including a signal peptide sequence, a peptidase S6 domain, a functional passenger domain, and a β-barrel autotransporter domain (Figure S2). At the nucleotide level the gene shows only partial identity with known sequences and presents a unique nucleotide sequence for the peptidase S6 and passenger domains. At the amino acid level, the autotransporter domain is identical to that of EspP and EspC from EHEC, while the passenger domain shares 33% sequence identity with EspP of *E. coli* O157:H7 strain Sakai (GenBank NP_052685.1) (Figure S2).

### A 9.7 kb element distinguishes pO26-CRL_125_ from pO111-CRL_115_

**Figure 4:**
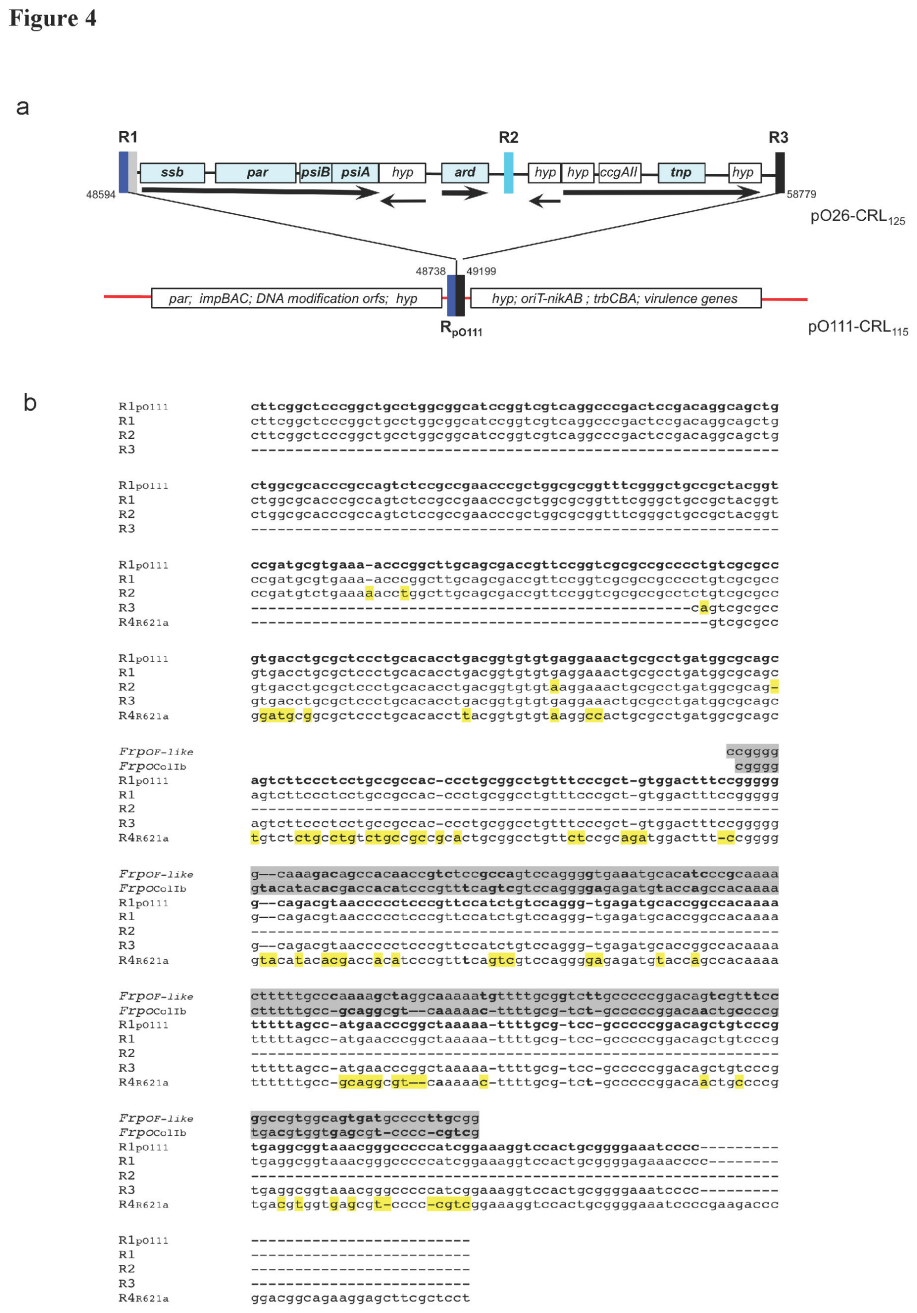
Schematic representation of the IncI leading region of pO26-CRL_125_. **a**. A 9.7 kb fragment, carrying genes involved in plasmid establishment in recipient cells during the early phases of conjugation, is the main distinguishing feature between pO26-CRL_125_ and pO111-CRL_115_. The insertion point in the backbone shared by pO26-CRL_125_ and pO111-CRL_115_ is indicated. The genes in the leading region are transcribed in the same direction (black arrows) from the single-stranded DNA promoter *Frpo* located in imperfect direct repeat sequences (R). The pO111-CRL_115_ sequence contains a R sequence (R_pO111_) identical to portions of both R1 and R3, but no leading region genes. Drawing not to scale. **b**. Alignment of R sequences found in pO26-CRL_125_ and pO111-CRL_115_ with *Frpo* containing sequences from IncI plasmids ColIb-P9 (GenBank NC_002122) and R64 (GenBank AP005147). The base differences between repeat sequences are highlighted in yellow. Alignment was obtained using the EMBO ClustalO online tool [36] (http://www.ebi.ac. uk/clustalO).

The 9,726 bp fragment present in pO26-CRL_125_ contains an IncI-associated genetic module implicated in the stable establishment in recipient cells following conjugation. This fragment is found in most IncI conjugative plasmids and contains an *ssb* gene coding for a single-stranded DNA (ssDNA) binding protein, a *parB* homolog for plasmid partitioning, *psiB* and *psiA* genes encoding SOS-response inhibition functions, and the conserved *ardA* and *ccgAII* orfs both with antirestriction function [42]. *ccgAII* is adjacent to a transposase encoding gene and four orfs with unknown function (Figure 4a; Table S1). Sequence analysis of the 9.7 kb region of pO26-CRL_125_ revealed the presence of three imperfect repeats (R1, R2, R3) with homology to the ssDNA promoter *Frpo* sequence (Figure 4b) [58,59]. The 9.7 kb module was entirely missing in the pO111-CRL_115_ backbone, but continuous sequence (R1_pO111_) almost identical to the pO26-CRL_125_ repeats was found at the point of insertion of the missing fragment (Figure 4a). The R1_pO111_ sequence in fact is identical to parts of both R1 (bases 1 to 239) and R3 (last 222 bases). These observations are consistent with the 9.7 kb fragment inserting via a double reciprocal crossover event. *Frpo*-containing sequences in sequenced IncI1 family plasmids are always associated with leading region genes exactly as seen in pO26-CRL_125_ (Figure 4a) or with minor variations, and are mostly located in proximity to DNA modifying genes and the *oriT* and *nikAB* orfs as seen in both pO26-CRL_125_ and pO111-CRL_115_ (Figure 4a). Variants may contain transposase or IS elements between repeats (pETEC_73 GenBank NC_009788.1; R621a GenBank NC_015965.1; p746 GenBank NC_014234.1) or the *hok*-*mok* post-segregational killing system replacing the *ccgAII* gene (p1658/97 GenBank NC_004998.1; p53638_75 GenBank NC_010720.1). Uniquely in pO111-CRL_115_, R1_pO111_ was not associated with any of the leading region components.

**Figure 5:**
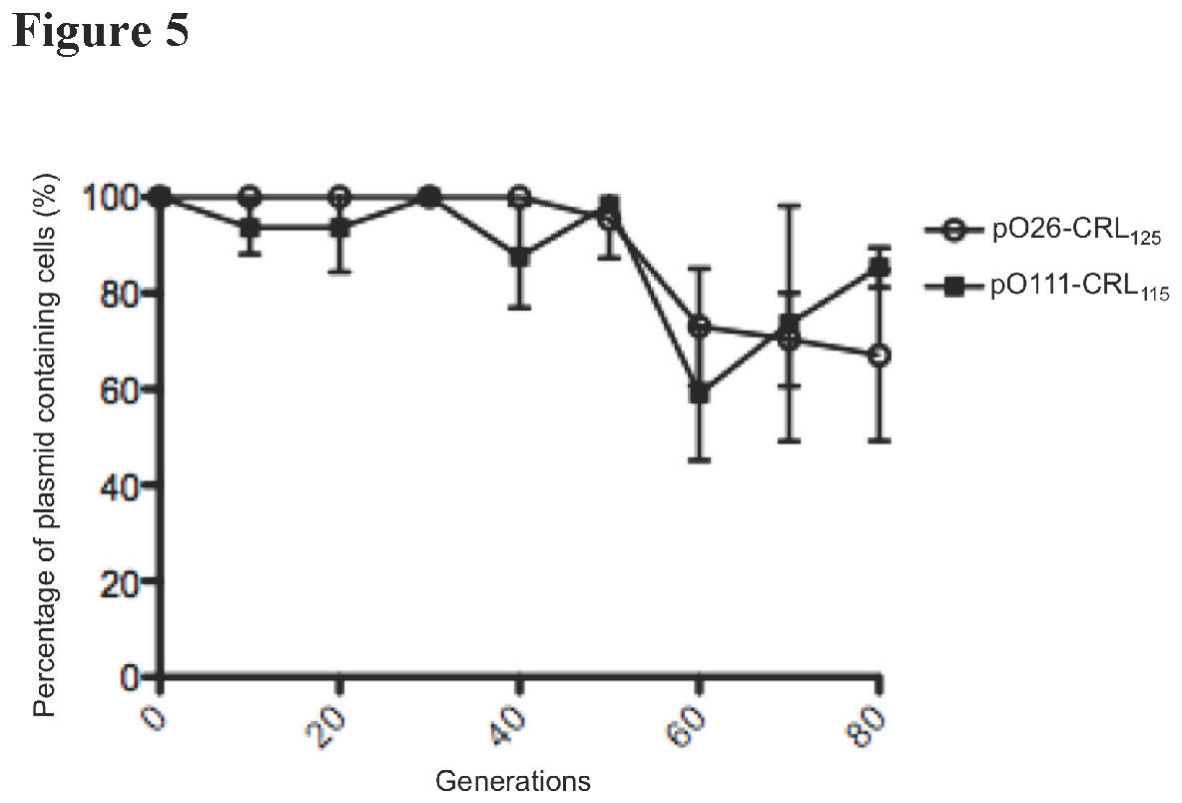
Stability curves. Stability of plasmids pO26-CRL_125_ and pO111-CRL_115_ was assessed over 80 generations by subculture in LB and selection on tetracycline supplemented solid media over 8 consecutive days. Both plasmids showed comparable high degrees of stability. Error bars indicate mean and standard deviation (*n* = 3).

In order to determine whether the differences in the sequence of pO111-CRL_115_ and pO26-CRL_125_ compromise the ability of each plasmid to mobilize and establish in recipient cells, plasmid conjugation and stability experiments were performed in parallel. In mating assays using *E. coli* JM109 Rif^r^Nal^r^ as recipient strain, both plasmids were able to self-transfer and showed comparable conjugation frequency (Table 2). In transformed *E. coli* TOP10 strains, no significant difference in stability between pO111-CRL_115_ and pO26-CRL_125_ was observed after 80 generations (Figure 5).

**Table 2:** Conjugal transfer of plasmids pO26-CRL_125_ and pO111-CRL_115_.

***E. coli* strains**	**No selection**	**Amp + Nal**	**Nal**	**Conjugation frequency**
TOP10 (pO26-CRL_125_)	1.2 x 10^9^	-	-	NA
TOP10 (pO111-CRL_115_)	1.1 x 10^9^	-	-	NA
JM109 Rif^r^Nal^r^	1.4 x 10^9^	-	3.8 x 10^9^	NA
TOP10 (pO26-CRL_125_) + JM109 Rif^r^Nal^r^	NA	7 x 10^5^	2.1 x 10^9^	3.3 x 10^-4^
TOP10 (pO111-CRL_115_) + JM109 Rif^r^Nal^r^	NA	1.3 x 10^6^	3.5 x 10^9^	3.7 x 10^-4^

NA, not applicable; Amp, ampicillin (100 μg/ml); Nal, rifampicin (30 μg/ml); -, no transconjugants detected.

## Discussion

The O26:H- EHEC strain O6877 is resistant to Ap, Km, Nm, Sm, Su, Tm and Tc and has been implicated as the causative agent of haemorrhagic colitis in an elderly patient [31]. We showed that two MDR plasmids, one a 124,908 bp IncZ plasmid pO26-CRL_125_ reported here, and a second 111,481 bp IncI1 plasmid pO26-CRL, renamed pO26-CRL_111_, reported earlier [26], co-exist in strain O6877. These plasmids carry different virulence gene combinations and plasmid incompatibility markers, but all genes encoding antibiotic resistance except for Tc are localized within the IR boundaries of identical derivate Tn*21* transposons. Five base pair direct repeats characteristic of Tn*21* transposition sites were found flanking the derivative transposon in the *traC* gene in pO26-CRL_111_ [26], but not in pO26-CRL_125_ or related plasmid pO111-CRL_115_. The transposition (*tnpA*) and resolvase (*tnpR*) genes and the IR_*tnp21*_ and IR_*mer21*_ inverted repeats are intact in pO26-CRL_125_. In EHEC strain O6877, it is likely that the derivate Tn*21* CRL originated in pO26-CRL_125_ and transposed into pO26-CRL_111_. STEC are readily isolated from the faeces of all ruminant species and many carry the enterohaemolysin gene *ehxA* on large plasmids that also carry other virulence genes [6,7,8,60]. The spread of the derivate Tn*21* transposon described in this study represents a mechanism by which virulence plasmids widely found in ruminant STEC populations can rapidly acquire resistance to multiple antibiotics in a single transposition event and is cause for serious concern. Little is known about the lateral transfer of EHEC plasmids and derivate mercury resistant transposons harboring CRL, between different pathotypes of *E. coli*. Recent studies provide evidence that food-producing animals represent one of the major reservoirs of *E. coli* causing urinary tract infections (UTI) [61]. Although antibiotics have limited use in the treatment of diarrheagenic *E. coli* infections, they are critical for management of extraintestinal infections.

In this study, we also reported the complete sequence of a large MDR plasmid, named pO111-CRL_115_, isolated from bovine O111 aEPEC strain D275. Bovine strain D275 is resistant to the same panel of antibiotics as human O26:H- EHEC strain O6877 and was positive for a diagnostic PCR we developed to detect atypical class 1 integrons [30]. The PCR, which has one primer in *intI1* and another in IS*26*, produced an 848 bp amplicon indicating that D275 carried a similar derivative Tn*21* transposon to that found in O26:H- EHEC strain O6877 [26,30]. The same 848 bp amplicon was previously identified in a group of serologically diverse multiply antibiotic resistant *E. coli* from cattle on farms located far apart on the east coast of Australia and in two isolates recovered from human UTI patients [30]. More recently, we also identified the 848 bp amplicon using the *intI1*-IS*26* PCR in several clinical isolates [62]. Composite transposons such as Tn*6029*/Tn*6026*, that carry *bla*_TEM_, *strAB*, and *sul2* genes flanked by IS*26*, are globally disseminated and contribute to the evolution of CRL [26,28,63,64].

In this study, we showed that pO111-CRL_115_ carries an identical copy of the derivate Tn*21* transposon found in pO26-CRL_125_ and pO26-CRL_111_. The CRL in pO26-CRL_125_ and pO111-CRL_115_ starts at the IR_*tnp21*_ and ends with IR_*tet1721*_. Tn*21* and Tn*1721* both belong to the Tn*3* family of transposons. Consequently, transposase-IR regions and their resolvases are functionally interchangeable and both generate 5 bp direct repeats at the site of transposition [65,66]. The structure of the CRL described here and the lack of 5 bp direct repeats suggest a complex evolutionary history involving laterally acquired DNA derived from disparate plasmids with different incompatibility, assembling within the IR boundaries via homologous recombination. These data reveal the critical role of derivate Tn*21* transposons in spreading CRL among diverse plasmids carrying different virulence gene combinations in *E. coli* belonging to different pathotypes. PCR targeting key loci that arise during the molecular evolution of CRL can be exploited to track mercury resistance transposons containing CRL and the plasmids that carry them. We provide direct evidence of the latter here.

Despite their different host origins, pO26-CRL_125_ and pO111-CRL_115_ are essentially identical plasmids. The main difference between them is a 9,726 bp fragment found only in pO26-CRL_125_. The 9,726 bp fragment, known as the IncI1 leading region, is characteristic of large IncI conjugative plasmids and contains a number of genes, including *ardA*, *psiAB*, and *ssb*, that function, often in a host-specific manner, during conjugation to protect single stranded DNA as it first enters the recipient cell [67,68,69]. The SOS response is known to influence the frequency of genetic rearrangement, particularly within integrons, as well as protect bacteria from external stresses in promiscuous environments [68,70]. Trimethoprim and β-lactam antibiotics are known to induce the SOS response [18]. The leading region genes are transiently expressed during conjugation and their expression is regulated by the specific *Frpo* promoter situated within repeat regions (R1, R2 and R3 in pO26-CRL_125_) [59]. To our knowledge, pO111-CRL_115_ is the first plasmid shown to completely lack the 9.7 kb fragment, but retain a *Frpo* sequence located exactly where the pO26-CRL_125_ leading region inserts. This suggests that the 9.7 kb fragment may actually be a novel mobile element. The presence of a putative transposase gene in the leading region sequence adds some weight to this speculation. We were unable to detect any difference in the conjugative efficiencies of pO111-CRL_115_ and pO26-CRL_125_ in standard laboratory conditions (Table 2). Further studies are needed to characterize the function of the 9,726 bp leading region and determine whether it can transpose independently.

Further comparative analysis of pO26-CRL_125_ and pO111-CRL_115_ sequences showed that regions of difference were invariably associated with features that either pertain to plasmid stability and may influence host range (leading region; *traH*) or confer flexibility to the plasmid backbone (repeats – RD1, RD2). The RD1 region, which separates the derivate Tn*21* transposon from ∆Tn*1721*, is essentially identical to the *oriV* of the IncP-1α plasmid pBS228, originally described in *Pseudomonas* [47]. While the *oriV*-∆Tn*1721* module is identical to one described in IncP-1β plasmid pB10 isolated from wastewater [51], the Tn*21*-RD1-ΔTn*1721* arrangement in pO26-CRL_125_ and pO111-CRL_115_ is unique. In both plasmids the *oriV* sequence found in Tn*21*-RD1-ΔTn*1721* is not expected to be functional as a replication feature because the *trfA* gene is missing [48]. Nonetheless, plasmids that carry *oriV*-type sequences are likely to gain an evolutionary advantage by virtue of the propensity of this region to serve as a hotspot for the acquisition of exogenous DNA [51,71].

pO26-CRL_125_ and pO111-CRL_115_ are conjugative, chimeric plasmids that contain genetic signatures common to both narrow host range IncI1 family plasmids and broad host range IncP family plasmids. The distinctive features in pO26-CRL_125_ and pO111-CRL_115_, including the unique shufflon, the IncZ replicon, unique toxin/antitoxin system and unique SPATE, and the regions of difference distinguishing the two plasmids, may be representative of adaptive responses to a lifestyle where the bacteria that house these plasmids move between bovine and human gastrointestinal tracts. This existence provides an opportunity for genes to be acquired laterally from microbial populations found in the soil and in wastewater ponds generated by food animal production. Members of the well characterized IncP-1 family are frequently isolated in the environment and carry readily mobilizable antibiotic resistance modules that play an important role in the lateral transfer of accessory genes between unrelated bacteria [50,72,73]. Conjugative IncI plasmids display a narrow host range and function as vehicles for the dissemination of antimicrobial resistance determinants in pathogenic *Enterobacteriaceae* [25]. The sequence differences observed in pO26-CRL_125_ and pO111-CRL_115_ suggest a likely role in bacterial adaptation to rapidly changing environments or host-specific recognition [74].

## Conclusions

This study and others [20] are indicative of how the problem of antibiotic resistance in humans may be linked to how antibiotics are used in food-animal production, aquaculture and horticulture. It is not possible to compartmentalize the problem because lateral gene transfer drives the movement of antibiotic resistance genes through these reservoirs and profoundly influences the delicate interplay between pathogenic and commensal bacterial populations [75]. Plasmids play a key role in the evolution of MDR *Enterobacteriaceae*, a key group in the struggle to curtail antibiotic resistance in the clinical environment [25]. Comprehensive analysis of complete sequences of plasmids carrying multiple antibiotic resistance genes is necessary to fully understand how CRL evolve and move through microbial populations in diverse settings.

## Supporting Information

Figure S1 **Features of the *oriT* region of plasmids pO26-CRL_125_ and pO111-CRL_115_.** a Inc RNAI sequence of pO26-CRL_125_ and pO111-CRL_115_ compared to that of other IncI family plasmids: EHEC plasmids pO26-CRL_111_ [26] and pO113 [56], and the prototype IncI1 plasmid R64 [S1]. Inc RNAI is a small antisense RNA essential for control of IncI plasmids replication. Due to the trans-acting nature of this type of replication control the Inc RNAI determines also the incompatibility of IncI family members. About 70 bases in length, it is encoded downstream of the *repYZ* genes and regulates copy number by binding to a complementary mRNA sequence in the 5´ end of *repZ* and silencing *repZ* [S2,S3]. The four Inc RNAI sequences shown here are not identical but present conserved features (underlined) conferring the specific secondary stem-loop structure involved in target binding. IncZ plasmids are compatible with IncI1 plasmids [S3]. b minimum *oriT* sequence of pO26-CRL_125_ and pO111-CRL_115_ compared to that of R64. The *oriT* minimal region is located immediately upstream of *nikA* in IncI1 plasmids such as R64. It can be identified by the presence of two sets of inverted repeats (17 bp in blue, and 8 bp in red) involved in protein binding [45]. In pO26-CRL_125_ and pO111-CRL_115_, the *oriT* sequence was immediately adjacent to the starting codon of *nikA* and contained both sets of repeats. The 8 bp repeats are identical to those of R64 while the 17 bp differ as it may be expected since the 17 bp inverted repeats constitute part of the recognised binding site for NikA and the NikA proteins of R64 and pO26-CRL_125_ and pO111-CRL_115_ share homology but are not identical.
(TIFF)

Figure S2 **Comparison of novel SPATE sequence identified in pO26-CRL_125_ and pO111-CRL_115_ with characterized EHEC SPATEs.** The novel SPATE sequence presents all the features characteristic of SPATEs: a conserved unusually long signal sequence (in bold magenta); a functional domain (underlined), containing a peptidase S6 domain (blue highlight) with a conserved serine protease motif GDSGS (bold, underlined), where the first S is the catalytic serine (red bold); and a very well conserved β-barrel autotransporter domain (green highlight). The functional domains are specific and show low homology to the characterized EHEC EspP from O157:H7 str Sakai *E. coli* (NP_052685.1), while the autotransporter domain is identical to that of EspP. Other conserved residues involved in protease activity are shown in red bold font.
(TIFF)

Table S1 **Open reading frames identified in the sequence of plasmids pO26-CRL_125_ and pO111-CRL_115_.**
(DOC)

References S1 (DOCX)

## References

[1] Nataro JP, Kaper JB (1998) Diarrheagenic *Escherichia* *coli* . Clin Microbiol Rev 11: 142-201. PubMed: 9457432. 9457432 10.1128/cmr.11.1.142 PMC121379

[2] McDaniel TK, Jarvis KG, Donnenberg MS, Kaper JB (1995) A genetic-locus of enterocyte effacement conserved among diverse enterobacterial pathogens. Proc Natl Acad Sci U S A 92: 1664-1668. doi:10.1073/pnas.92.5.1664. PubMed: 7878036. 7878036 PMC42580

[3] Boerlin P, McEwen SA, Boerlin-Petzold F, Wilson JB, Johnson RP et al. (1999) Associations between virulence factors of Shiga toxin-producing *Escherichia* *coli* and disease in humans. J Clin Microbiol 37: 497-503. PubMed: 9986802. 9986802 10.1128/jcm.37.3.497-503.1999 PMC84443

[4] Karch H, Tarr PI, Bielaszewska M (2005) Enterohaemorrhagic Escherichia coli in human medicine. Int J Med Microbiol 295: 405-418. doi:10.1016/j.ijmm.2005.06.009. PubMed: 16238016. 16238016

[5] Moura RA, Sircili MP, Leomil L, Matté MH, Trabulsi LR et al. (2009) Clonal relationship among atypical enteropathogenic *Escherichia* *coli* strains isolated from different animal species and humans. Appl Environ Microbiol 75: 7399-7408. doi:10.1128/AEM.00636-09. PubMed: 19801470. 19801470 PMC2786407

[6] Hornitzky MA, Mercieca K, Bettelheim KA, Djordjevic SP (2005) Bovine feces from animals with gastrointestinal infections are a source of serologically diverse atypical enteropathogenic *Escherichia* *coli* and Shiga toxin-producing *E.* *coli* strains that commonly possess intimin. Appl Environ Microbiol 71: 3405-3412. doi:10.1128/AEM.71.7.3405-3412.2005. PubMed: 16000742. 16000742 PMC1168988

[7] Djordjevic SP, Ramachandran V, Bettelheim KA, Vanselow BA, Holst P et al. (2004) Serotypes and virulence gene profiles of shiga toxin-producing *Escherichia* *coli* strains isolated from feces of pasture-fed and lot-fed sheep. Appl Environ Microbiol 70: 3910-3917. doi:10.1128/AEM.70.7.3910-3917.2004. PubMed: 15240263. 15240263 PMC444789

[8] Hornitzky MA, Vanselow BA, Walker K, Bettelheim KA, Corney B et al. (2002) Virulence properties and serotypes of Shiga toxin-producing *Escherichia* *coli* from healthy Australian cattle. Appl Environ Microbiol 68: 6439-6445. doi:10.1128/AEM.68.12.6439-6445.2002. PubMed: 12450875. 12450875 PMC134377

[9] Bettelheim KA (2007) The non-O157 shiga-toxigenic (verocytotoxigenic) *Escherichia* *coli*; under-rated pathogens. Crit Rev Microbiol 33: 67-87. doi:10.1080/10408410601172172. PubMed: 17453930. 17453930

[10] Rangel JM, Sparling PH, Crowe C, Griffin PM, Swerdlow DL (2005) Epidemiology of *Escherichia* *coli* O157:H7 outbreaks, United States, 1982-2002. Emerg Infect Dis 11: 603-609. doi:10.3201/eid1104.040739. PubMed: 15829201. 15829201 PMC3320345

[11] Vally H, Hall G, Dyda A, Raupach J, Knope K et al. (2012) Epidemiology of Shiga toxin producing *Escherichia* *coli* in Australia, 2000-2010. BMC Public Health 12: 63. doi:10.1186/1471-2458-12-63. PubMed: 22264221. 22264221 PMC3398300

[12] Johnson KE, Thorpe CM, Sears CL (2006) The emerging clinical importance of non-O157 Shiga toxin-producing *Escherichia* *coli* . Clin Infect Dis 43: 1587-1595. doi:10.1086/509573. PubMed: 17109294. 17109294

[13] Zimmerhackl LB, Rosales A, Hofer J, Riedl M, Jungraithmayr T et al. (2010) Enterohemorrhagic Escherichia coli O26:H11-associated hemolytic uremic syndrome: bacteriology and clinical presentation. Semin Thromb Hemost 36: 586-593. doi:10.1055/s-0030-1262880. PubMed: 20865635. 20865635

[14] Mellmann A, Bielaszewska M, Köck R, Friedrich AW, Fruth A et al. (2008) Analysis of collection of hemolytic uremic syndrome-associated enterohemorrhagic Escherichia coli. Emerg Infect Dis 14: 1287-1290. doi:10.3201/eid1408.071082. PubMed: 18680658. 18680658 PMC2600372

[15] Pollock KG, Bhojani S, Beattie TJ, Allison L, Hanson M et al. (2011) Highly virulent *Escherichia* *coli* O26, Scotland. Emerg Infect Dis 17: 1777-1779. doi:10.3201/eid1709.110199. PubMed: 21888827. 21888827 PMC3322084

[16] Shiomi M, Togawa M, Fujita K, Murata R (1999) Effect of early oral fluoroquinolones in hemorrhagic colitis due to *Escherichia* *coli* O157:H7. Pediatr Int 41: 228-232. doi:10.1046/j.1442-200X.1999.4121038.x. PubMed: 10221035. 10221035

[17] Safdar N, Said A, Gangnon RE, Maki DG (2002) Risk of hemolytic uremic syndrome after antibiotic treatment of *Escherichia* *coli* O157:H7 enteritis: a meta-analysis. JAMA 288: 996-1001. doi:10.1001/jama.288.8.996. PubMed: 12190370. 12190370

[18] Hastings PJ, Rosenberg SM, Slack A (2004) Antibiotic-induced lateral transfer of antibiotic resistance. Trends Microbiol 12: 401-404. doi:10.1016/j.tim.2004.07.003. PubMed: 15337159. 15337159

[19] Kimmitt PT, Harwood CR, Barer MR (2000) Toxin gene expression by shiga toxin-producing *Escherichia* *coli*: the role of antibiotics and the bacterial SOS response. Emerg Infect Dis 6: 458-465. doi:10.3201/eid0605.000503. PubMed: 10998375. 10998375 PMC2627954

[20] Muniesa M, Hammerl JA, Hertwig S, Appel B, Brüssow H (2012) Shiga toxin-producing *Escherichia* *coli* O104:H4: a new challenge for microbiology. Appl Environ Microbiol 78: 4065-4073. doi:10.1128/AEM.00217-12. PubMed: 22504816. 22504816 PMC3370534

[21] Lee JH (2009) Antimicrobial resistance of *Escherichia* *coli* O26 and O111 isolates from cattle and their characteristics. Vet Microbiol 135: 401-405. doi:10.1016/j.vetmic.2008.09.076. PubMed: 18992996. 18992996

[22] Valat C, Haenni M, Saras E, Auvray F, Forest K et al. (2012) CTX-M-15 extended-spectrum beta-lactamase in a shiga toxin-producing *Escherichia* *coli* isolate of serotype O111:H8. Appl Environ Microbiol 78: 1308-1309. doi:10.1128/AEM.06997-11. PubMed: 22156432. 22156432 PMC3273006

[23] Buvens G, Bogaerts P, Glupczynski Y, Lauwers S, Piérard D (2010) Antimicrobial resistance testing of verocytotoxin-producing *Escherichia* *coli* and first description of TEM-52 extended-spectrum beta-lactamase in serogroup O26. Antimicrob Agents Chemother 54: 4907-4909. doi:10.1128/AAC.00551-10. PubMed: 20733038. 20733038 PMC2976167

[24] Partridge SR (2011) Analysis of antibiotic resistance regions in Gram-negative bacteria. FEMS Microbiol Rev 35: 820-855. doi:10.1111/j.1574-6976.2011.00277.x. PubMed: 21564142. 21564142

[25] Carattoli A (2009) Resistance plasmid families in Enterobacteriaceae. Antimicrob Agents Chemother 53: 2227-2238. doi:10.1128/AAC.01707-08. PubMed: 19307361. 19307361 PMC2687249

[26] Venturini C, Beatson SA, Djordjevic SP, Walker MJ (2010) Multiple antibiotic resistance gene recruitment onto the enterohemorrhagic Escherichia coli virulence plasmid. FASEB J 24: 1160-1166. doi:10.1096/fj.09-144972. PubMed: 19917674. 19917674

[27] Doublet B, Praud K, Weill FX, Cloeckaert A (2009) Association of IS*26*-composite transposons and complex In4-type integrons generates novel multidrug resistance loci in *Salmonella* genomic island 1. J Antimicrob Chemother 63: 282-289. PubMed: 19074421. 19074421 10.1093/jac/dkn500

[28] Cain AK, Liu X, Djordjevic SP, Hall RM (2010) Transposons related to Tn*1696* in IncHI2 plasmids in multiply antibiotic resistant *Salmonella* *enterica* serovar Typhimurium from Australian animals. Microb Drug Resist 16: 197-202. doi:10.1089/mdr.2010.0042. PubMed: 20701539. 20701539

[29] Perron GG, Lee AE, Wang Y, Huang WE, Barraclough TG (2012) Bacterial recombination promotes the evolution of multi-drug-resistance in functionally diverse populations. Proc Biol Sci 279: 1477-1484. doi:10.1098/rspb.2011.1933. PubMed: 22048956. 22048956 PMC3282345

[30] Dawes FE, Kuzevski A, Bettelheim KA, Hornitzky MA, Djordjevic SP et al. (2010) Distribution of class 1 integrons with IS*26*-mediated deletions in their 3'-conserved segments in *Escherichia* *coli* of human and animal origin. PLOS ONE 5: e12754. doi:10.1371/journal.pone.0012754. PubMed: 20856797. 20856797 PMC2939871

[31] Bettelheim KA, Hornitzky MA, Djordjevic SP, Kuzevski A (2003) Antibiotic resistance among verocytotoxigenic *Escherichia* *coli* (VTEC) and non-VTEC isolated from domestic animals and humans. J Med Microbiol 52: 155-162. doi:10.1099/jmm.0.04903-0. PubMed: 12543922. 12543922

[32] Walker MJ, Pemberton JM (1987) Construction of a transposon containing a gene for polygalacturonate trans-eliminase from *Klebsiella* *oxytoca* . Arch Microbiol 146: 390-395. doi:10.1007/BF00410941. PubMed: 3034186. 3034186

[33] Aziz RK, Bartels D, Best AA, DeJongh M, Disz T et al. (2008) The RAST Server: rapid annotations using subsystems technology. BMC Genomics 9: 75. doi:10.1186/1471-2164-9-75. PubMed: 18261238. 18261238 PMC2265698

[34] Altschul SF, Gish W, Miller W, Myers EW, Lipman DJ (1990) Basic local alignment search tool. J Mol Biol 215: 403-410. doi:10.1016/S0022-2836(05)80360-2. PubMed: 2231712. 2231712

[35] Siguier P, Perochon J, Lestrade L, Mahillon J, Chandler M (2006) ISfinder: the reference centre for bacterial insertion sequences. Nucleic Acids Res 34: D32-D36. doi:10.1093/nar/gkj014. PubMed: 16381877. 16381877 PMC1347377

[36] Sievers F, Wilm A, Dineen DG, Gibson TJ, Karplus K et al. (2011) Fast, scalable generation of high-quality protein multiple sequence alignments using Clustal Omega. Mol Syst Biol 7: 539 PubMed: 21988835. 21988835 10.1038/msb.2011.75 PMC3261699

[37] Stothard P, Wishart DS (2005) Circular genome visualization and exploration using CGView. Bioinformatics 21: 537-539. doi:10.1093/bioinformatics/bti054. PubMed: 15479716. 15479716

[38] De Gelder L, Ponciano JM, Joyce P, Top EM (2007) Stability of a promiscuous plasmid in different hosts: no guarantee for a long-term relationship. Microbiol 153: 452-463. doi:10.1099/mic.0.2006/001784-0. PubMed: 17259616. 17259616

[39] Keen NT, Tamaki S, Kobayashi D, Trollinger D (1988) Improved broad-host-range plasmids for DNA cloning in gram-negative bacteria. Gene 70: 191-197. doi:10.1016/0378-1119(88)90117-5. PubMed: 2853689. 2853689

[40] Ogura Y, Ooka T, Iguchi A, Toh H, Asadulghani M et al. (2009) Comparative genomics reveal the mechanism of the parallel evolution of O157 and non-O157 enterohemorrhagic Escherichia coli. Proc Natl Acad Sci U S A 106: 17939-17944. doi:10.1073/pnas.0903585106. PubMed: 19815525. 19815525 PMC2764950

[41] Praszkier J, Wei T, Siemering K, Pittard J (1991) Comparative-analysis of the replication regions of IncB, IncK, and IncZ plasmids. J Bacteriol 173: 2393-2397. PubMed: 1706708. 1706708 10.1128/jb.173.7.2393-2397.1991 PMC207792

[42] Takahashi H, Shao M, Furuya N, Komano T (2011) The genome sequence of the incompatibility group Iγ plasmid R621a: evolution of IncI plasmids. Plasmid 66: 112-121. doi:10.1016/j.plasmid.2011.06.004. PubMed: 21763721. 21763721

[43] Gerdes K, Christensen SK, Løbner-Olesen A (2005) Prokaryotic toxin-antitoxin stress response loci. Nat Rev Microbiol 3: 371-382. doi:10.1038/nrmicro1147. PubMed: 15864262. 15864262

[44] Gotfredsen M, Gerdes K (1998) The *Escherichia* *coli* *relBE* genes belong to a new toxin-antitoxin gene family. Mol Microbiol 29: 1065-1076. doi:10.1046/j.1365-2958.1998.00993.x. PubMed: 9767574. 9767574

[45] Furuya N, Komano T (1991) Determination of the nick site at *oriT* of IncI1 plasmid R64 - global similarity of *oriT* structures of IncI1 and IncP plasmids. J Bacteriol 173: 6612-6617. PubMed: 1917882. 1917882 10.1128/jb.173.20.6612-6617.1991 PMC208999

[46] Komano T (1999) Shufflons: multiple inversion systems and integrons. Annu Rev Genet 33: 171-191. doi:10.1146/annurev.genet.33.1.171. PubMed: 10690407. 10690407

[47] Haines AS, Jones K, Batt SM, Kosheleva IA, Thomas CM (2007) Sequence of plasmid pBS228 and reconstruction of the IncP-1α phylogeny. Plasmid 58: 76-83. doi:10.1016/j.plasmid.2007.01.001. PubMed: 17320955. 17320955

[48] Adamczyk M, Jagura-Burdzy G (2003) Spread and survival of promiscuous IncP-1 plasmids. Acta Biochim Pol 50: 425-453. PubMed: 12833168. 12833168

[49] Allmeier H, Cresnar B, Greck M, Schmitt R (1992) Complete nucleotide sequence of Tn*1721*: gene organization and a novel gene product with features of a chemotaxis protein. Gene 111: 11-20. doi:10.1016/0378-1119(92)90597-I. PubMed: 1312499. 1312499

[50] Eikmeyer F, Hadiati A, Szczepanowski R, Wibberg D, Schneiker-Bekel S et al. (2012) The complete genome sequences of four new IncN plasmids from wastewater treatment plant effluent provide new insights into IncN plasmid diversity and evolution. Plasmid 68: 13-24. doi:10.1016/j.plasmid.2012.01.011. PubMed: 22326849. 22326849

[51] Schlüter A, Heuer H, Szczepanowski R, Forney LJ, Thomas CM et al. (2003) The 64508 bp IncP-1β antibiotic multiresistance plasmid pB10 isolated from a waste-water treatment plant provides evidence for recombination between members of different branches of the IncP-1β group. Microbiol 149: 3139-3153. doi:10.1099/mic.0.26570-0. 14600226

[52] Johnson TJ, Shepard SM, Rivet B, Danzeisen JL, Carattoli A (2011) Comparative genomics and phylogeny of the IncI1 plasmids: a common plasmid type among porcine enterotoxigenic Escherichia coli. Plasmid 66: 144-151. doi:10.1016/j.plasmid.2011.07.003. PubMed: 21843549. 21843549

[53] Sen D, Van der Auwera GA, Rogers LM, Thomas CM, Brown CJ et al. (2011) Broad-Host-Range plasmids from agricultural soils have IncP-1 backbones with diverse accessory genes. Appl Environ Microbiol 77: 7975-7983. doi:10.1128/AEM.05439-11. PubMed: 21948829. 21948829 PMC3209000

[54] Sota M, Tsuda M, Yano H, Suzuki H, Forney LJ et al. (2007) Region-specific insertion of transposons in combination with selection for high plasmid transferability and stability accounts for the structural similarity of IncP-1 plasmids. J Bacteriol 189: 3091-3098. doi:10.1128/JB.01906-06. PubMed: 17277066. 17277066 PMC1855856

[55] Konisky J (1982) Colicins and other bacteriocins with established modes of action. Annu Rev Microbiol 36: 125-144. doi:10.1146/annurev.mi.36.100182.001013. PubMed: 6184011. 6184011

[56] Leyton DL, Sloan J, Hill RE, Doughty S, Hartland EL (2003) Transfer region of pO113 from enterohemorrhagic Escherichia coli: similarity with R64 and identification of a novel plasmid-encoded autotransporter, EpeA. Infect Immun 71: 6307-6319. doi:10.1128/IAI.71.11.6307-6319.2003. PubMed: 14573650. 14573650 PMC219559

[57] Dautin N (2010) Serine Protease Autotransporters of Enterobacteriaceae (SPATEs): biogenesis and function. Toxins (Basel) 2: 1179-1206. doi:10.3390/toxins2061179. PubMed: 22069633. 22069633 PMC3153244

[58] Masai H, Arai K (1997) Frpo: a novel single-stranded DNA promoter for transcription and for primer RNA synthesis of DNA replication. Cell 89: 897-907. doi:10.1016/S0092-8674(00)80275-5. PubMed: 9200608. 9200608

[59] Bates S, Roscoe RA, Althorpe NJ, Brammar WJ, Wilkins BM (1999) Expression of leading region genes on IncI1 plasmid ColIb-P9: genetic evidence for single-stranded DNA transcription. Microbiol 145: 2655-2662. PubMed: 10537187. 10.1099/00221287-145-10-2655 10537187

[60] Ramachandran V, Brett K, Hornitzky MA, Dowton M, Bettelheim KA et al. (2003) Distribution of intimin subtypes among *Escherichia* *coli* isolates from ruminant and human sources. J Clin Microbiol 41: 5022-5032. doi:10.1128/JCM.41.11.5022-5032.2003. PubMed: 14605134. 14605134 PMC262460

[61] Nordstrom L, Liu CM, Price LB (2013) Foodborne urinary tract infections: a new paradigm for antimicrobial-resistant foodborne illness. Front Microbiol 4: 1-6. PubMed: 23346082. 23508293 10.3389/fmicb.2013.00029 PMC3589730

[62] Djordjevic SP, Stokes HW, Chowdhury Roy P (2013) Mobile elements, zoonotic pathogens and commensal bacteria: conduits for the delivery of resistance genes into humans, production animals and soil microbiota. Front Microbiol 4: 1-12. PubMed: 23346082. 23641238 10.3389/fmicb.2013.00086 PMC3639385

[63] Szczepanowski R, Braun S, Riedel V, Schneiker S, Krahn I, Pühler A, Schlüter A (2005) The 120 592 bp IncF plasmid pRSB107 isolated from a sewage-treatment plant encodes nine different antibiotic-resistance determinants, two iron-acquisition systems and other putative virulence-associated functions. Microbiol 151: 1095-1111. doi:10.1099/mic.0.27773-0. 15817778

[64] Labar AS, Millman JS, Ruebush E, Opintan JA, Bishar RA et al. (2012) Regional dissemination of a trimethoprim-resistance gene cassette via a successful transposable element. PLOS ONE 7(5): e38142. doi:10.1371/journal.pone.0038142. PubMed: 22666464. 22666464 PMC3364232

[65] Grinsted J, de la Cruz F, Altenbuchner J, Schmitt R (1982) Complementation of transposition of *tnpA* mutants of Tn*3*, Tn*21*, Tn*501*, and Tn*1721* . Plasmid 8: 276-286. doi:10.1016/0147-619X(82)90065-8. PubMed: 6294711. 6294711

[66] Halford SE, Jordan SL, Kirkbride EA (1985) The resolvase protein from the transposon Tn*21* . Mol Gen Genet 200: 169-175. doi:10.1007/BF00383331. PubMed: 2993810. 2993810

[67] Bagdasarian M, Bailone A, Angulo JF, Scholz P, Bagdasarian M et al. (1992) PsiB, an anti-SOS protein, is transiently expressed by the F-Sex factor during its transmission to an *Escherichia* *coli* K-12 recipient. Mol Microbiol 6: 885-893. doi:10.1111/j.1365-2958.1992.tb01539.x. PubMed: 1318487. 1318487

[68] Baharoglu Z, Bikard D, Mazel D (2010) Conjugative DNA transfer induces the bacterial SOS response and promotes antibiotic resistance development through integron activation. PLOS Genet 6(10): e1001165 PubMed: 20975940. 20975940 10.1371/journal.pgen.1001165 PMC2958807

[69] Althorpe NJ, Chilley PM, Thomas AT, Brammar WJ, Wilkins BM (1999) Transient transcriptional activation of the IncI1 plasmid anti-restriction gene (*ardA*) and SOS inhibition gene (*psiB*) early in conjugating recipient bacteria. Mol Microbiol 31: 133-142. doi:10.1046/j.1365-2958.1999.01153.x. PubMed: 9987116. 9987116

[70] Guerin E, Cambray G, Sanchez-Alberola N, Campoy S, Erill I et al. (2009) The SOS response controls integron recombination. Science 324: 1034-1034. doi:10.1126/science.1172914. PubMed: 19460999. 19460999

[71] Pinyon JL, Hall RM (2011) Evolution of IncP-1α plasmids by acquisition of antibiotic and mercuric ion resistance transposons. Microb Drug Resist 17: 339-343. doi:10.1089/mdr.2010.0196. PubMed: 21476866. 21476866

[72] Heuer H, Schmitt H, Smalla K (2011) Antibiotic resistance gene spread due to manure application on agricultural fields. Curr Opin Microbiol 14: 236-243. doi:10.1016/j.mib.2011.04.009. PubMed: 21546307. 21546307

[73] Smalla K, Haines AS, Jones K, Krögerrecklenfort E, Heuer H et al. (2006) Increased abundance of IncP-1β plasmids and mercury resistance genes in mercury-polluted river sediments: first discovery of IncP-1β plasmids with a complex *mer* transposon as the sole accessory element. Appl Environ Microbiol 72: 7253-7259. doi:10.1128/AEM.00922-06. PubMed: 16980416. 16980416 PMC1636140

[74] McKenzie GJ, Harris RS, Lee PL, Rosenberg SM (2000) The SOS response regulates adaptive mutation. Proc Natl Acad Sci U S A 97: 6646-6651. doi:10.1073/pnas.120161797. PubMed: 10829077. 10829077 PMC18688

[75] Ochman H, Lawrence JG, Groisman EA (2000) Lateral gene transfer and the nature of bacterial innovation. Nature 405: 299-304. doi:10.1038/35012500. PubMed: 10830951. 10830951

