# Supplementary figures and images for: Sequences of Two Related Multiple Antibiotic Resistance Virulence Plasmids Sharing a Unique IS26-Related Molecular Signature Isolated from Different Escherichia coli Pathotypes from Different Hosts
Source: PLoS One. 2013 Nov 4;8(11):e78862. doi: 10.1371/journal.pone.0078862 (PMC3817090; doi:10.1371/journal.pone.0078862)

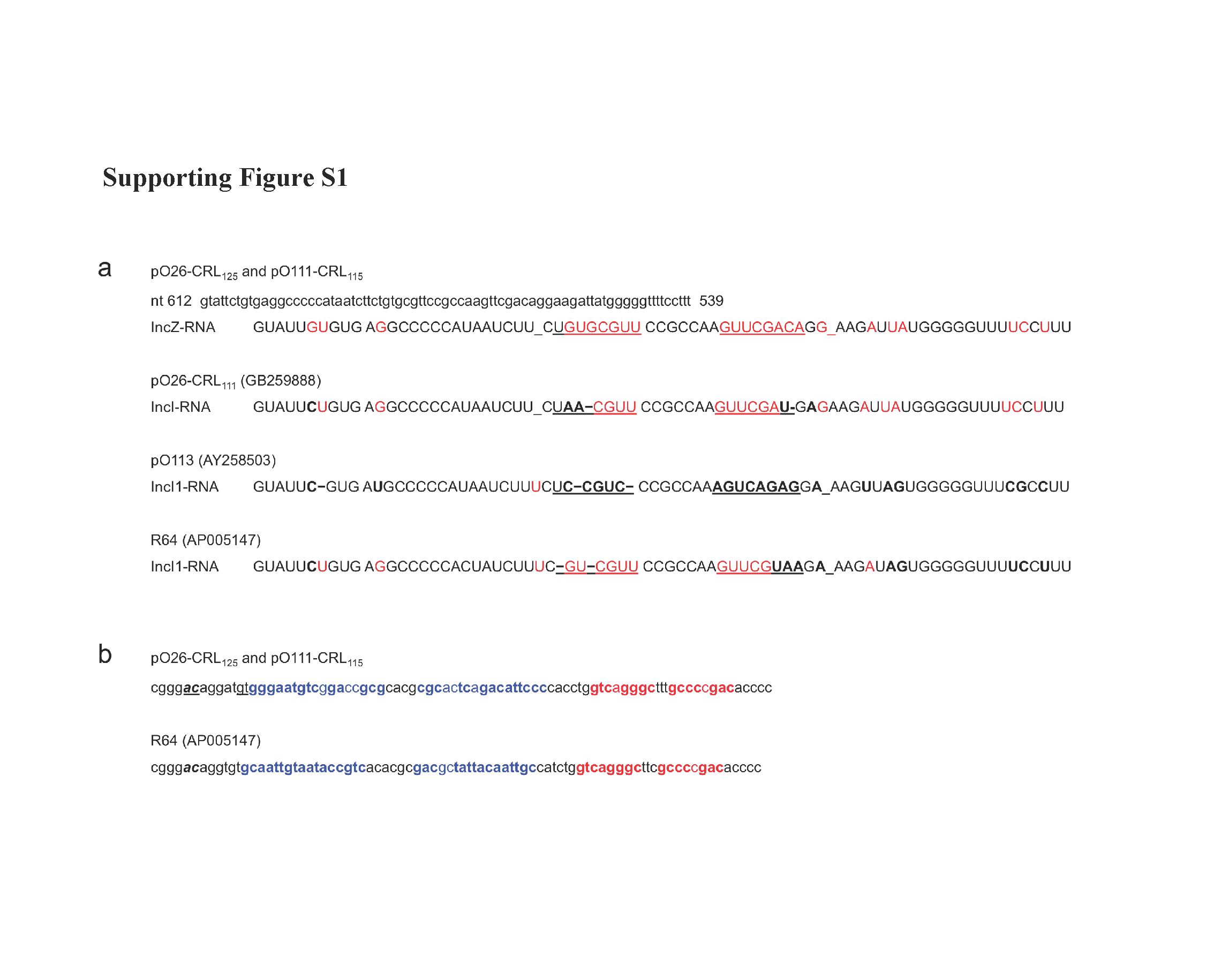

Supplement: Figure S1 — Features of the oriT region of plasmids pO26-CRL125 and pO111-CRL115. a Inc RNAI sequence of pO26-CRL125 and pO111-CRL115 compared to that of other IncI family plasmids: EHEC plasmids pO26-CRL111 [26] and pO113 [56], and the prototype IncI1 plasmid R64 [S1]. Inc RNAI is a small antisense RNA essential for control of IncI plasmids replication. Due to the trans-acting nature of this type of replication control the Inc RNAI determines also the incompatibility of IncI family members. About 70 bases in length, it is encoded downstream of the repYZ genes and regulates copy number by binding to a complementary mRNA sequence in the 5´ end of repZ and silencing repZ [S2,S3]. The four Inc RNAI sequences shown here are not identical but present conserved features (underlined) conferring the specific secondary stem-loop structure involved in target binding. IncZ plasmids are compatible with IncI1 plasmids [S3]. b minimum oriT sequence of pO26-CRL125 and pO111-CRL115 compared to that of R64. The oriT minimal region is located immediately upstream of nikA in IncI1 plasmids such as R64. It can be identified by the presence of two sets of inverted repeats (17 bp in blue, and 8 bp in red) involved in protein binding [45]. In pO26-CRL125 and pO111-CRL115, the oriT sequence was immediately adjacent to the starting codon of nikA and contained both sets of repeats. The 8 bp repeats are identical to those of R64 while the 17 bp differ as it may be expected since the 17 bp inverted repeats constitute part of the recognised binding site for NikA and the NikA proteins of R64 and pO26-CRL125 and pO111-CRL115 share homology but are not identical. (TIFF) [file pone.0078862.s001.tiff]

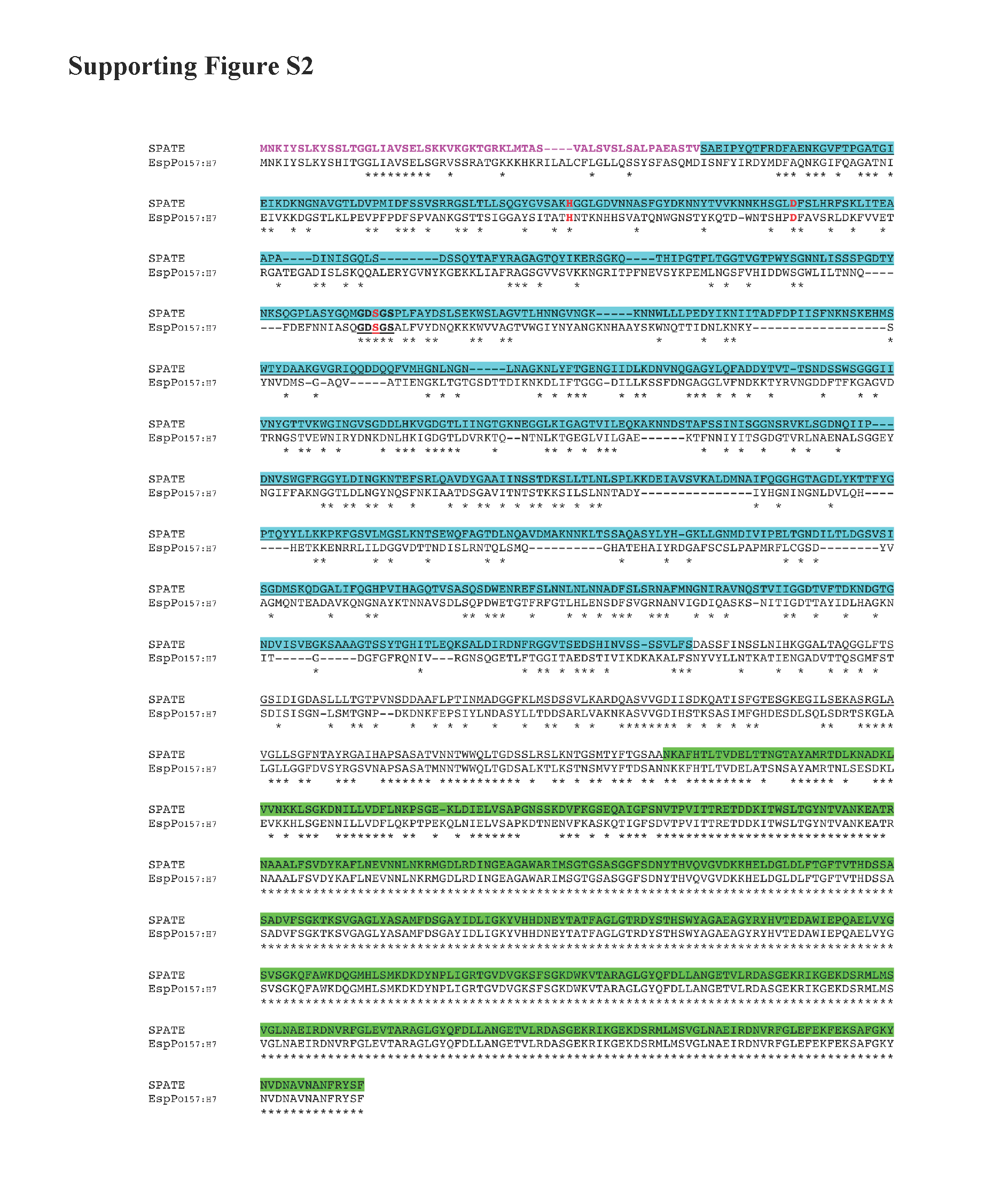

Supplement: Figure S2 — Comparison of novel SPATE sequence identified in pO26-CRL125 and pO111-CRL115 with characterized EHEC SPATEs. The novel SPATE sequence presents all the features characteristic of SPATEs: a conserved unusually long signal sequence (in bold magenta); a functional domain (underlined), containing a peptidase S6 domain (blue highlight) with a conserved serine protease motif GDSGS (bold, underlined), where the first S is the catalytic serine (red bold); and a very well conserved β-barrel autotransporter domain (green highlight). The functional domains are specific and show low homology to the characterized EHEC EspP from O157:H7 str Sakai E. coli (NP_052685.1), while the autotransporter domain is identical to that of EspP. Other conserved residues involved in protease activity are shown in red bold font. (TIFF) [file pone.0078862.s002.tiff]
