# Supplementary material for: Sequences of Two Related Multiple Antibiotic Resistance Virulence Plasmids Sharing a Unique IS26-Related Molecular Signature Isolated from Different Escherichia coli Pathotypes from Different Hosts
Source: PLoS One. 2013 Nov 4;8(11):e78862. doi: 10.1371/journal.pone.0078862 (PMC3817090; doi:10.1371/journal.pone.0078862)
Supplement: Table S1 — Open reading frames identified in the sequence of plasmids pO26-CRL125 and pO111-CRL115. (DOC) [file pone.0078862.s003.doc]

**Supporting Table S1**

| **Table S1. Open reading frames identified in the sequence of plasmids pO26-CRL_125_ and pO111-CRL_115_.** | | | | | | | | | |
| --- | --- | --- | --- | --- | --- | --- | --- | --- | --- |
| **pO26-CRL_125_** | | | **pO111-CRL_115_** | | | **Strand** | **Name** | **Function** | **% nt^†^ identity (GenBank)** |
| **orf *** | **start** | **stop** | **orf *** | **start** | **stop** |  |  |  |  |
| 1 | 626 | 716 | 1 | 626 | 716 | + | *repY* | replication | 100 pEI545 (M93064) |
| 2 | 703 | 1078 | 2 | 703 | 1078 | + | *repZ* | replication | 100 pEI545 (M93064) |
| 3 | 1925 | 2053 | 3 | 1925 | 2053 | + | *hyp* | hypthetical protein | 96 pCoo (CR942285) |
| 4 | 2270 | 2437 | 4 | 2270 | 2437 | + | *hyp* | hypthetical protein | 95 pCoo (CR942285) |
| 5 | 2524 | 2772 | 5 | 2524 | 2772 | + | *stbD* | replicon stabilization | 94 pAPEC-O1-ColBM (DQ381420) |
| 6 | 2772 | 3056 | 6 | 2772 | 3056 | + | *stbE* | replicon stabilization toxin | no significant similarity |
| 7 | 3592 | 3467 | 7 | 3592 | 3467 | - | *hyp* | hypthetical protein | no significant similarity |
| 8 | 4249 | 4028 | 8 | 4249 | 4028 | - | *hyp* | hypthetical protein | 100 pColIb-P9 (AB021078) |
| 9 | 4269 | 4868 | 9 | 4269 | 4868 | - | *yafB* | activator of osmoprotectant transporter | 99 pColIb-P9 (AB021078) |
| 10 | 5462 | 5222 | 10 | 5462 | 5222 | - | Δ*yagA* | hypothetical protein | 87 pColIb-P9 (AB021078) |
| 11 | 8462 | 5499 | 11 | 8462 | 5499 | - | *tnpA* | Tn*21* transposase | 100 pO26-CRL (GQ259888) |
| 12 | 9025 | 8468 | 12 | 9025 | 8468 | - | *tnpR* | Tn*21* resolvase | 100 pO26-CRL (GQ259888) |
| 13 | 9735 | 9154 | 13 | 9735 | 9154 | - | *tnpM* | Tn*21* modulator | 100 pO26-CRL (GQ259888) |
| 14 | 10717 | 9707 | 14 | 10717 | 9707 | - | *intI1* | integrase IntI1 | 100 pO26-CRL (GQ259888) |
| 15 | 10873 | 11343 | 15 | 10873 | 11343 | + | *dfrA5* | dihydrofolate reductase | 100 pO26-CRL (GQ259888) |
| 16 | 12203 | 11501 | 16 | 12203 | 11501 | - | IS*26 tnp* | IS*26* transposase | 100 pO26-CRL (GQ259888) |
| 17 | 12362 | 12601 | 17 | 12362 | 12601 | + | Δ*res* | truncated res | 100 pO26-CRL (GQ259888) |
| 18 | 12787 | 13644 | 18 | 12787 | 13644 | + | *blaTEM_1_* | β-lactamase | 100 pO26-CRL (GQ259888) |
| 19 | 14024 | 13884 | 19 | 14024 | 13884 | - | Δ*tnpB* | 3'-part of *tnpB* | 100 pO26-CRL (GQ259888) |
| 20 | 14233 | 14946 | 20 | 14233 | 14946 | + | *IS26 tnp* | IS*26* transposase | 100 pO26-CRL (GQ259888) |
| 21 | 15011 | 15715 | 21 | 15011 | 15715 | + | Δ*repA* | replication protein | 100 pO26-CRL (GQ259888) |
| 22 | 15705 | 16553 | 22 | 15705 | 16553 | + | *repC* | replication protein | 100 pO26-CRL (GQ259888) |
| 23 | 16864 | 17676 | 23 | 16864 | 17676 | + | *sul2* | dihydropteroate synthase | 100 pO26-CRL (GQ259888) |
|  |  |  |  |  |  |  |  |  |  |
|  |  |  |  |  |  |  |  |  |  |
| *table continues next page* | | | | | | | | | |
| *Table S1 continued* | | |  | | |  |  |  |  |
| **pO26-CRL_125_** | | | **pO111-CRL_115_** | | | **Strand** | **Name** | **Function** | **% nt^†^ identity (GenBank)** |
| **orf *** | **start** | **stop** | **orf *** | **start** | **stop** |  |  |  |  |
| 24 | 17740 | 18539 | 24 | 17740 | 18539 | + | *strA* | aminoglycoside 3'-phosphotransferase | 100 pO26-CRL (GQ259888) |
| 25 | 18539 | 19375 | 25 | 18539 | 19375 | + | *strB* | aminoglycoside 3'-phosphotransferase | 100 pO26-CRL (GQ259888) |
| 26 | 20155 | 19442 | 26 | 20155 | 19442 | - | IS*26 tnp* | IS*26* transposase | 100 pO26-CRL (GQ259888) |
| 27 | 20348 | 21106 | 27 | 20348 | 21106 | + | *aphA1* | aminoglycoside 3'-phosphotransferase | 100 pO26-CRL (GQ259888) |
| 28 | 22003 | 21302 | 28 | 22003 | 21302 | - | IS*26 tnp* | IS*26* transposase | 100 pO26-CRL (GQ259888) |
| 29 | 23318 | 22050 | 29 | 23318 | 22050 | - | Δ*tniA* | TniA putative transposase | 100 pO26-CRL (GQ259888) |
| 30 | 24100 | 23396 | 30 | 24100 | 23396 | - | *urf2* | unknown function | 100 pO26-CRL (GQ259888) |
| 31 | 24333 | 24100 | 31 | 24333 | 24100 | - | *merE* | mercuric transport protein | 100 pO26-CRL (GQ259888) |
| 32 | 24692 | 24333 | 32 | 24692 | 24333 | - | *merD* | mercuric resistance operon coregulator | 100 pO26-CRL (GQ259888) |
| 33 | 26404 | 24713 | 33 | 26404 | 24713 | - | *merA* | mercuric ion reductase | 100 pO26-CRL (GQ259888) |
| 34 | 26917 | 26459 | 34 | 26917 | 26459 | - | *merC* | mercuric transport protein, | 100 pO26-CRL (GQ259888) |
| 35 | 27189 | 26917 | 35 | 27189 | 26917 | - | *merP* | periplasmic mercury binding protein | 100 pO26-CRL (GQ259888) |
| 36 | 27553 | 27206 | 36 | 27553 | 27206 | - | *merT* | mercuric transport protein | 100 pO26-CRL (GQ259888) |
| 37 | 27625 | 28056 | 37 | 27625 | 28056 | + | *merR* | mercuric resistance operon regulatory protein | 100 pO26-CRL (GQ259888) |
| 38 | 28902 | 29141 | 38 | 29062 | 29301 | + | *hyp* | relaxase /helicase | 100 pBS228 (AM261760) |
| 39 | 29853 | 29179 | 39 | 30013 | 29339 | - | *tetR* | transcriptional regulator | 100 pBS228 (AM261760) |
| 40 | 29857 | 31128 | 40 | 30017 | 31288 | + | *tetA* | tetracycline efflux protein | 100 pBS228 (AM261760) |
| 41 | 32047 | 31166 | 41 | 32207 | 31326 | - | *pecM* | permease of the drug/metabolite transporter | 100 pBS228 (AM261760) |
|  |  |  |  |  |  |  |  |  |  |
|  | | |  |  |  |  |  |  | *table continues next page* |
| *Table S1 continued* | | |  |  |  |  |  |  |  |
| **pO26-CRL_125_** | | | **pO111-CRL_115_** | | | **Strand** | **Name** | **Function** | **% nt^†^ identity (GenBank)** |
| **orf *** | **start** | **stop** | **orf *** | **start** | **stop** |  |  |  |  |
| 42 | 32577 | 32188 | 42 | 32737 | 32348 | - | *hyp* | amidases related to nicotinamidase | 100 pB10 (AJ564903) |
| 43 | 32536 | 34329 | 43 | 32696 | 34489 | + | *tnpA* | Tn*1721* transposase | 100 pB10 (AJ564903) |
| 44 | 34366 | 36072 | 44 | 34526 | 36232 | + | Δ*cib* | colicin production protein | 99 pColIb-P9 (AB021078.1) |
| 45 | 36437 | 36093 | 45 | 36597 | 36253 | - | *cim* | colicin-Ib immunity protein | 99 pColIb-P9 (AB021078.1) |
| 46 | 37084 | 36926 | 46 | 37244 | 37086 | - | *ydeA* | hypothetical protein | 96 pColIb-P9 (AB021078.1) |
| 47 | 37702 | 37523 | 47 | 37846 | 37667 | - | *hyp* | hypothetical protein | 100 p3521 (GU256641) |
| 48 | 38448 | 37825 | 48 | 38592 | 37969 | - | *parA* | chromosome partitioning | 100 p3521 (GU256641) |
| 49 | 39923 | 38655 | 49 | 40067 | 38799 | - | *impB* | ImpB UV protection and mutation | 98 p3521 (GU256641) |
| 50 | 40360 | 39926 | 50 | 40504 | 40070 | - | *impA* | ImpA UV protection and mutation | 99 p3521 (GU256641) |
| 51 | 40605 | 40360 | 51 | 40749 | 40501 | - | *impC* | ImpC | 99 p3521 (GU256641) |
| 52 | 41628 | 43349 | 52 | 41772 | 43493 | + | *hyp* | retron-type reverse transcriptase | 100 pO157 (AB011549) |
| 53 | 43429 | 44328 | 53 | 43573 | 44472 | + | *hyp* | hypothetical protein | 99 pO113 (AY258503) |
| 54 | 44608 | 44486 | 54 | 44752 | 44630 | - | *hyp* | hypothetical protein | 100 pO113 (AY258503) |
| 55 | 44716 | 45396 | 55 | 44860 | 45540 | + | *hap* | adenine-specific methyltransferase | 98 pO113 (AY258503) |
| 56 | 45400 | 45621 | 56 | 45544 | 45765 | + | *hyp* | hypothetical protein | 95 pColIb-P9 (AB021078) |
| 57 | 45635 | 46066 | 57 | 45779 | 46210 | + | *ycgB* | hypothetical protein | 94 pColIb-P9 (AB021078) |
| 58 | 46115 | 46888 | 58 | 46259 | 47032 | + | *hyp* | hypothetical protein | 95 pO157 (AB011549) |
| 59 | 47228 | 47010 | 59 | 47372 | 47154 | - | *hyp* | hypothetical protein | 99 pColIb-P9 (AB021078) |
| 60 | 47309 | 47731 | 60 | 47453 | 47875 | + | *yfeB* | putative antirestriction protein | 97 p3521 (GU256641) |
| 61 | 47781 | 48197 | 61 | 47925 | 48341 | + | *yfeC* | hypothetical protein | 98 p3521 (GU256641) |
| 62 | 48200 | 48388 | 62 | 48344 | 48532 | + | *hyp* | hypothetical protein | 97 p3521 (GU256641) |
| 63 | 49160 | 49687 | - | NA | NA | + | *ssb* | single-stranded DNA-binding protein | 99 p3521 (GU256641) |
| 64 | 50031 | 51992 | - | NA | NA | + | *parB-like* | putative cytoplasmic protein | 99 p3521 (GU256641) |
|  | | |  | | |  |  |  | *table continues next page* |
| *Table S1 continued* | | |  | | |  |  |  |  |
| **pO26-CRL_125_** | | | **pO111-CRL_115_** | | | **Strand** | **Name** | **Function** | **% nt^†^ identity (GenBank)** |
| **orf *** | **start** | **stop** | **orf *** | **start** | **stop** |  |  |  |  |
| 65 | 52050 | 52481 | - | NA | NA | + | *psiB* | SOS-inhibition | 99 p3521 (GU256641) |
| 66 | 52481 | 53197 | - | NA | NA | + | *psiA* | SOS-inhibition | 99 p3521 (GU256641) |
| 67 | 53197 | 53790 | - | NA | NA | + | *eaa* | Eaa protein | 98 p3521 (GU256641) |
| 68 | 54022 | 54753 | - | NA | NA | + | *ardA* | antirestriction protein | 100 pColIb-P9 (AB021078) |
| 69 | 55487 | 55918 | - | NA | NA | + | *hyp* | hypothetical protein | 97 p3521 (GU256641) |
| 70 | 55918 | 56280 | - | NA | NA | + | *ydfB* | hypothetical protein | 96 p3521 (GU256641) |
| 71 | 56249 | 56860 | - | NA | NA | + | *ccgAII* | conserved hypothetical protein | 91 p3521 (GU256641) |
| 72 | 56857 | 57780 | - | NA | NA | + | *tnp* | transposase 31 family | 99 pO113 (AY258503) |
| 73 | 58038 | 58286 | - | NA | NA | + | *hyp* | hypothetical protein | 97 p3521 (GU256641) |
| 74 | 59710 | 58865 | 63 | 50129 | 49355 | - | *ydiA* | Z1226 protein | 96 p3521 (GU256641) |
| 75 | 60133 | 59798 | 64 | 50552 | 50217 | - | *yggA* | hypothetical protein | 98 p3521 (GU256641) |
| 76 | 60366 | 60695 | 65 | 50785 | 51114 | + | *nikA* | nickel ABC transporter, nickel-binding protein | 99 pO113 (AY258503) |
| 77 | 60710 | 63418 | 66 | 51129 | 53837 | + | *nikB* | conjugative DNA transfer | 99 pO113 (AY258503) |
| 78 | 63753 | 63920 | 67 | 54172 | 54339 | + | *hyp* | hypothetical protein | 100 pO157 (AB011549) |
| 79 | 64018 | 64179 | 68 | 54437 | 54598 | - | *hyp* | putative iron-regulated virulence protein | 99 pO113 (AY258503) |
| 80 | 64601 | 64308 | 69 | 55020 | 54727 | - | *hyp* | hypothetical protein | 98 pO113 (AY258503) |
| 81 | 67018 | 64715 | 70 | 57437 | 55134 | - | *trbC* | hypothetical protein | 99 pO113 (AY258503) |
| 82 | 68117 | 66999 | 71 | 58536 | 57418 | - | *trbB* | conjugative transfer | 99 pO113 (AY258503) |
| 83 | 69403 | 68114 | 72 | 59822 | 58533 | - | *trbA* | conjugative transfer | 99 pO113 (AY258503) |
| 84 | 70450 | 70139 | 73 | 60869 | 60558 | - | *hyp* | neurotensin receptor R8 | 98 pO113 (AY258503) |
| 85 | 70669 | 70478 | 74 | 61088 | 60897 | - | *hyp* | hypothetical protein | 100 pO113 (AY258503) |
| 86 | 71044 | 70682 | 75 | 61463 | 61101 | - | *hyp* | hypothetical protein | 98 pO113 (AY258503) |
| 87 | 71246 | 71055 | 76 | 61665 | 61474 | - | *hyp* | hypothetical protein | 99 pO113 (AY258503) |
| 88 | 71226 | 71387 | 77 | 61645 | 61806 | + | *hyp* | hypothetical protein | 99 pO113 (AY258503) |
| 89 | 75713 | 71550 | 78 | 66132 | 61969 | - | *SPATE* | serine protease autotransporter enterotoxin | 99 pO113 (AY258503) β-barrel only |
| 90 | 76510 | 76043 | 79 | 66929 | 66462 | - | *hyp* | putative nuclease FinO-like | 98 pO113 (AY258503) |
|  | | |  | | |  |  |  | *table continues next page* |
| *Table S1 continued* | | |  | | |  |  |  |  |
| **pO26-CRL_125_** | | | **pO111-CRL_115_** | | | **Strand** | **Name** | **Function** | **% nt^†^ identity (GenBank)** |
| **orf *** | **start** | **stop** | **orf *** | **start** | **stop** |  |  |  |  |
| 91 | 76669 | 77292 | 80 | 67088 | 67711 | + | *hyp* | hypothetical protein | 97 pO113 (AY258503) |
| 92 | 77854 | 78003 | 81 | 68273 | 68422 | + | *hyp* | hypothetical protein | 95 pECOED (CU928147) |
| 93 | 78035 | 79570 | 82 | 68454 | 69989 | + | *hyp* | hypothetical protein | 99 pO113 (AY258503) |
| 94 | 79663 | 80025 | 83 | 70444 | 700082 | - | *hyp* | hypothetical protein | 99 pO113 (AY258503) |
| 95 | 80382 | 80075 | 84 | 70801 | 70494 | - | *pndA* | post-segregational killing | 98 pO113 (AY258503) |
| 96 | 80237 | 80389 | 85 | 70656 | 70808 | + | *pndC* | post-segregational killing | 97 pECOED (CU928147) |
| 97 | 80547 | 80386 | 86 | 70966 | 70805 | - | *hyp* | hypothetical protein | 96 pECOED (CU928147) |
| 98 | 81855 | 81211 | 87 | 72274 | 71630 | - | *excA* | surface exclusion protein | 88 pECOED (CU928147) |
| 99 | 84107 | 81942 | 88 | 74526 | 72361 | - | *traY* | IncI1 conjugative transfer | 94 pO113 (AY258503) |
| 100 | 84644 | 84180 | 89 | 75063 | 74599 | - | *traX* | IncI1 conjugative transfer | 100 pO113 (AY258503) |
| 101 | 85951 | 84746 | 90 | 76370 | 75165 | - | *traW* | IncI1 conjugative transfer | 99 pO113 (AY258503) |
| 102 | 86529 | 85909 | 91 | 76948 | 76328 | - | *traV* | IncI1 conjugative transfer | 99 pO113 (AY258503) |
| 103 | 89573 | 86529 | 92 | 79992 | 76948 | - | *traU* | IncI1 conjugative transfer | 99 pO113 (AY258503) |
| 104 | 90218 | 90024 | 93 | 80637 | 80443 | - | *hyp* | hypothetical protein | no significant similarity |
| 105 | 90696 | 90316 | 94 | 81115 | 80735 | - | *hyp* | unique hypothetical protein | 95 pO113 (AY258503) |
| 106 | 91055 | 90696 | 95 | 81474 | 81115 | - | *hyp* | unique hypothetical protein | 95 pO113 (AY258503) |
| 107 | 91827 | 91114 | 96 | 82246 | 81533 | - | *traT* | IncI1 conjugative transfer | 99 pO113 (AY258503) |
| 108 | 92098 | 91847 | 97 | 82517 | 82266 | - | *traS* | IncI1 conjugative transfer | 99 pO113 (AY258503) |
| 109 | 92553 | 92155 | 98 | 82972 | 82574 | - | *traR* | IncI1 conjugative transfer | 95 pO113 (AY258503) |
| 110 | 93132 | 92602 | 99 | 83551 | 83021 | - | *traQ* | IncI1 conjugative transfer | 99 pO113 (AY258503) |
|  |  |  |  |  |  |  |  |  |  |
|  |  |  |  |  |  |  |  |  | *table continues next page* |
| *Table S1 continued* | | |  | | |  |  |  |  |
| **pO26-CRL_125_** | | | **pO111-CRL_115_** | | | **Strand** | **Name** | **Function** | **% nt^†^ identity (GenBank)** |
| **orf *** | **start** | **stop** | **orf *** | **start** | **stop** |  |  |  |  |
| 111 | 93842 | 93129 | 100 | 84261 | 83548 | - | *traP* | IncI1 conjugative transfer | 99 pO113 (AY258503) |
| 112 | 95170 | 93839 | 101 | 85589 | 84258 | - | *traO* | IncI1 conjugative transfer | 99 pO113 (AY258503) |
| 113 | 96148 | 95174 | 102 | 86567 | 85593 | - | *traN* | IncI1 conjugative transfer | 99 pO113 (AY258503) |
| 114 | 96854 | 96159 | 103 | 87273 | 86578 | - | *traM* | IncI1 conjugative transfer | 99 pO113 (AY258503) |
| 115 | 97216 | 96866 | 104 | 87635 | 87285 | - | *traL* | IncI1 conjugative transfer | 99 pO113 (AY258503) |
| 116 | 101249 | 97233 | 105 | 91668 | 87652 | - | *sogL* | IncI1 conjugative transfer DNA primase | 99 pO113 (AY258503) |
| 117 | 101603 | 101313 | 106 | 92022 | 91732 | - | *traK* | IncI1 conjugative transfer | 100 pO113 (AY258503) |
| 118 | 102748 | 101600 | 107 | 93167 | 92019 | - | *traJ* | IncI1 conjugative transfer | 99 pO113 (AY258503) |
| 119 | 103568 | 102732 | 108 | 93987 | 93151 | - | *traI* | IncI1 conjugative transfer | 99 pO113 (AY258503) |
| 120 | 103882 | 103565 | 109 | 94436 | 93984 | - | *traH* | IncI1conjugative transfer | 99 pO113 (AY258503) |
| 121 | 104101 | 103931 | 110 | 94655 | 94485 | - | *hyp* | hypothetical protein | 99 pO113 (AY258503) |
| 122 | 104443 | 104241 | 111 | 95997 | 94795 | - | *traF* | IncI1 conjugative transfer | 99 pO113 (AY258503) |
| 123 | 106353 | 105532 | 112 | 96907 | 96086 | - | *traE* | IncI1 conjugative transfer | 99 pO113 (AY258503) |
| 124 | 106943 | 106623 | 113 | 97487 | 97167 | + | *hyp* | hypothetical protein | 99 pO113 (AY258503) |
| 125 | 107198 | 107034 | 114 | 97742 | 97578 | - | *hyp* | unique hypothetical protein | 99 pO113 (AY258503) |
| 126 | 108535 | 107405 | 115 | 99079 | 97949 | - | *rci* | shufflon-specific DNA recombinase | 92 ICESe4 (FR686852) |
| 127 | 108862 | 108720 | 116 | 99406 | 99264 | + | *pilV-3'* | shufflon protein | 81 pSH146_65 (JN983044) |
| 128 | 109445 | 109149 | 117 | 99989 | 99693 | - | *pilV-3'* | shufflon protein B | 97 pO113 (AY258503) |
| 129 | 110513 | 109527 | 118 | 101057 | 100071 | - | *pilV* | IncI1 pilus adhesin N terminus | 98 pO113 (AY258503) |
|  |  |  |  |  |  |  |  |  | *table continues next page* |
| *Table S1 continued* | | |  | | |  |  |  |  |
| **pO26-CRL_125_** | | | **pO111-CRL_115_** | | | **Strand** | **Name** | **Function** | **% nt^†^ identity (GenBank)** |
| **orf *** | **start** | **stop** | **orf *** | **start** | **stop** |  |  |  |  |
| 130 | 111153 | 110518 | 119 | 101697 | 101062 | - | *pilU* | IncI1 conjugative transfer peptidase/N-methyltransferase | 97 pO113 (AY258503) |
| 131 | 111645 | 111172 | 120 | 102189 | 101716 | - | *pilT* | IncI1 conjugative transfer | 100 pO113 (AY258503) |
| 132 | 112235 | 111699 | 121 | 102779 | 102243 | - | *pilS* | IncI1 conjugative transfer prepilin | 99 pO113 (AY258503) |
| 133 | 113409 | 112309 | 122 | 103953 | 102853 | - | *pilR* | IncI1 conjugative transfer | 99 pO113 (AY258503) |
| 134 | 114919 | 113411 | 123 | 105463 | 103955 | - | *pilQ* | IncI1 conjugative transfer ATPase | 99 pO113 (AY258503) |
| 135 | 115456 | 115004 | 124 | 106000 | 105548 | - | *pilP* | IncI1 assembly | 99 pO113 (AY258503) |
| 136 | 116741 | 115446 | 125 | 107285 | 105990 | - | *pilO* | IncI1 pilus assembly | 99 pO113 (AY258503) |
| 137 | 117241 | 116762 | 126 | 107785 | 107306 | - | *ΔpilN* | IncI1 conjugative transfer lipoprotein C-terminus | 99 pO113 (AY258503) |
| 138 | 118381 | 117238 | 127 | 108925 | 107782 | - | *ΔpilN* | IncI1 conjugative transfer lipoprotein N-terminus | 99 pO113 (AY258503) |
| 139 | 118849 | 118412 | 128 | 109393 | 108956 | - | *pilM* | IncI1 conjugative transfer | 99 pO113 (AY258503) |
| 140 | 119399 | 118854 | 129 | 109943 | 109398 | - | Δ*pilL* | IncI1 conjugative transfer | 99 pO113 (AY258503) |
| 141 | 119921 | 119435 | 130 | 110465 | 109979 | - | Δ*pilL* | IncI1 conjugative transfer | 99 pO113 (AY258503) |
| 142 | 120434 | 120078 | 131 | 110978 | 110622 | - | *hyp* | hypothetical protein | 99 pO113 (AY258503) |
| 143 | 120772 | 120530 | 132 | 111316 | 111074 | - | *pilI* | IncI1 conjugative transfer | 99 pO113 (AY258503) |
| 144 | 122547 | 120853 | 133 | 113091 | 111397 | - | *ygiK* | putative membrane protein | 99 pO113 (AY258503) |
| 145 | 123194 | 122574 | 134 | 113738 | 113118 | - | *ygiJ* | hypothetical protein | 99 pO113 (AY258503) |
| 146 | 124126 | 123461 | 135 | 114670 | 114005 | - | *traC* | IncI1 conjugative transfer | 99 pO113 (AY258503) |
|  |  |  |  |  |  |  |  |  | *table continues next page* |
| *Table S1 continued* | | |  |  |  |  |  |  |  |
| **pO26-CRL_125_** | | | **pO111-CRL_115_** | | | **Strand** | **Name** | **Function** | **% nt^†^ identity (GenBank)** |
| **orf *** | **start** | **stop** | **orf *** | **start** | **stop** |  |  |  |  |
| 147 | 124908 | 124267 | 136 | 115452 | 114811 | - | *traB* | IncI1 conjugative transfer transcription antiterminator | 99 pO113 (AY258503) |
| *orf, open reading frame; **^†^**nt, nucleotide. | | | | | | | | | |
