# Supplementary material for: Sequences of Two Related Multiple Antibiotic Resistance Virulence Plasmids Sharing a Unique IS26-Related Molecular Signature Isolated from Different Escherichia coli Pathotypes from Different Hosts
Source: PLoS One. 2013 Nov 4;8(11):e78862. doi: 10.1371/journal.pone.0078862 (PMC3817090; doi:10.1371/journal.pone.0078862)
Supplement: References S1 — (DOCX) [file pone.0078862.s004.docx]

**Supporting References S1**

S1. Sampei G, Furuya N, Tachibana K, Saitou Y, Suzuki T, et al. (2010) Complete genome sequence of the incompatibility group I1 plasmid R64. Plasmid 64: 92-103.

S2. del Solar G, Giraldo R, [Ruiz-Echevarría](http://mmbr.asm.org/search?author1=Mar%C3%ADa+Jes%C3%BAs+Ruiz-Echevarr%C3%ADa&sortspec=date&submit=Submit) GMJ, et al. (1998) Replication and control of circular bacterial plasmids. Microbiol Mol Biol Rev 62: 434-464.

S3. Praszkier J, Pittard AJ (2005) Control of replication in I-complex plasmids. Plasmid 53: 97-112.
